# NuA4 Lysine Acetyltransferase Complex Contributes to Phospholipid Homeostasis in *Saccharomyces cerevisiae*

**DOI:** 10.1534/g3.117.041053

**Published:** 2017-04-28

**Authors:** Louis Dacquay, Annika Flint, James Butcher, Danny Salem, Michael Kennedy, Mads Kaern, Alain Stintzi, Kristin Baetz

**Affiliations:** * Ottawa Institute of Systems Biology, University of Ottawa, Ontario K1H 8M5, Canada; † Department of Biochemistry, Microbiology, and Immunology, University of Ottawa, Ontario K1H 8M5, Canada; ‡ Department of Cellular and Molecular Medicine, University of Ottawa, Ontario K1H 8M5, Canada

**Keywords:** triacylglycerols, steryl esters, inositol/choline responsive elements (ICREs), *FAS1*/*FAS2*, cerulenin

## Abstract

Actively proliferating cells constantly monitor and readjust their metabolic pathways to ensure the replenishment of phospholipids necessary for membrane biogenesis and intracellular trafficking. In *Saccharomyces cerevisiae*, multiple studies have suggested that the lysine acetyltransferase complex NuA4 plays a role in phospholipid homeostasis. For one, NuA4 mutants induce the expression of the inositol-3-phosphate synthase gene, *INO1*, which leads to excessive accumulation of inositol, a key metabolite used for phospholipid biosynthesis. Additionally, NuA4 mutants also display negative genetic interactions with *sec14-1^ts^*, a mutant of a lipid-binding gene responsible for phospholipid remodeling of the Golgi. Here, using a combination of genetics and transcriptional profiling, we explore the connections between NuA4, inositol, and Sec14. Surprisingly, we found that NuA4 mutants did not suppress but rather exacerbated the growth defects of *sec14-1^ts^* under inositol-depleted conditions. Transcriptome studies reveal that while loss of the NuA4 subunit *EAF1* in *sec14-1^ts^* does derepress *INO1* expression, it does not derepress all inositol/choline-responsive phospholipid genes, suggesting that the impact of Eaf1 on phospholipid homeostasis extends beyond inositol biosynthesis. In fact, we find that NuA4 mutants have impaired lipid droplet levels and through genetic and chemical approaches, we determine that the genetic interaction between *sec14-1^ts^* and NuA4 mutants potentially reflects a role for NuA4 in fatty acid biosynthesis. Altogether, our work identifies a new role for NuA4 in phospholipid homeostasis.

Cellular processes, such as proliferation and intracellular trafficking, depend on a vast network of proteins involved in regulating phospholipid metabolism (Figure 1). Membrane phospholipid composition is, in part, established by highly conserved families of lipid-binding proteins that, as their name suggests, bind cellular lipids to facilitate lipid transport, modulate enzymatic activity, and even regulate the transcription of metabolic genes (reviewed in Henry *et al.* 2012; Olkkonen 2013; Jackson and Bouvet 2014; Tripathi *et al.* 2014). Phospholipid metabolism can also be fine-tuned by signaling pathways such as post-translational modifications. Indeed, it has recently emerged that lysine acetylation contributes to lipid metabolism by regulating both gene expression and the activity of metabolic enzymes (reviewed in Drazic *et al.* 2016; Menzies *et al.* 2016).

**Figure 1:**
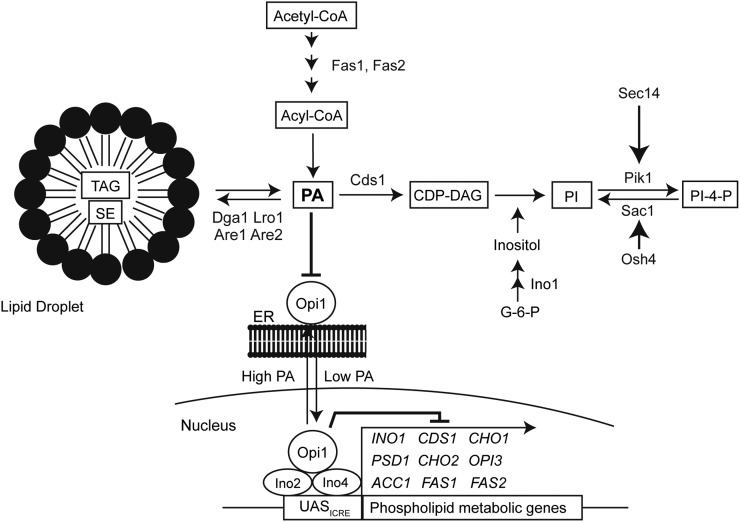
Overview of phospholipid metabolism. Key lipid metabolic proteins mentioned in the text are indicated, transcription factors/repressors are in circles, and lipids species are indicated in black rectangles. CDP-DAG, cytidine diphosphate diacylglycerol; PA, phosphatidic acid; PI, phosphatidylinositol; PI-4-P, phosphatidylinositol-4-phosphate; TAG, triacylglycerol; SE, steryl ester.

Several genome-wide genetic screens performed in *Saccharomyces cerevisiae* have revealed interactions between the highly conserved lysine acetyltransferase (KAT) complex NuA4 and phospholipid metabolic genes, suggesting a role for NuA4 in phospholipid homeostasis. NuA4 is a 13-subunit KAT complex containing the essential catalytic domain Esa1 (reviewed in Doyon and Cote 2004) and held together by the scaffolding protein Eaf1 (Auger *et al.* 2008; Mitchell *et al.* 2008). One role for NuA4 is the regulation of chromatin remodeling and gene transcription through the acetylation of histones H4 and H2A-Z (reviewed in Lu *et al.* 2009), and growing evidence indicates that NuA4 also targets nonhistone proteins (reviewed in Downey and Baetz 2016).

Interestingly, several functional genomic screens have determined that NuA4 subunit mutants display an excessive accumulation of inositol, also called an Opi- phenotype (Hancock *et al.* 2006; Salas-Santiago and Lopes 2014). Opi1 is a lipid-binding protein that represses transcription of target genes including the inositol-3-phosphate synthase, *INO1*, as well as other phospholipid metabolic genes containing inositol/choline-responsive elements (ICREs) within their promoters (Greenberg *et al.* 1982; Hirsch and Henry 1986; White *et al.* 1991). These genes are derepressed under inositol-depleted conditions through a mechanism involving the relocation of Opi1 from the nucleus to the endoplasmic reticulum, where it binds to phosphatidic acid (Loewen *et al.* 2003, 2004). Deleting *OPI1* leads to the overexpression of *INO1*, which in turn causes an excessive accumulation of inositol, referred to as the Opi- phenotype (Greenberg *et al.* 1982). High-throughput genomic screens have identified many other mutants that derepress *INO1* transcription and cause excessive accumulation of inositol, including mutants of the NuA4 complex (*EAF1*, *EAF3*, *EAF5*, *EAF7*, *YAF9*, and *ESA1*) (Hancock *et al.* 2006; Salas-Santiago and Lopes 2014). Interestingly, despite binding to its promoter, NuA4 is not required for the transcriptional activation of *INO1* (Suka *et al.* 2001; Konarzewska *et al.* 2012), suggesting that the Opi- phenotype of NuA4 mutants is due to an uncharacterized role for NuA4 in the regulation of ICRE-containing genes as well as phospholipid homeostasis. NuA4 subunit mutants are unique among Opi- mutants as they also negatively interact with a mutant of the phospholipid-remodeling protein Sec14, providing an opportunity to dissect the particular role of NuA4 in phospholipid metabolism.

Sec14 is an essential phospholipid-binding protein that coordinates the metabolism of phosphatidylinositol-4-phosphate (PI-4-P) with phosphatidylcholine (PC) at the Golgi to create a lipid environment necessary for trafficking events (reviewed in Ghosh and Bankaitis 2011). Sec14-deficient mutants cause multiple effects related to phospholipid metabolism, including increased intracellular PC (McGee *et al.* 1994; Xie *et al.* 2001) and decreased PI-4-P (Hama *et al.* 1999; Li *et al.* 2002; Schaaf *et al.* 2008). However, Sec14-deficient mutants also exhibits growth defects under inositol-depleted conditions (Patton-Vogt *et al.* 1997), which is associated with an inability to derepress *INO1* expression leading to decreased inositol biosynthesis under conditions lacking an exogenous source of inositol (Culbertson and Henry 1975; Villa-Garcia *et al.* 2011). Synthetic genetic screens identified a mutant of the NuA4 complex, *eaf7*Δ, as one of several mutants that display a growth defect in combination with the temperature-sensitive mutant *sec14-G266N* (*sec14-1^ts^*) (Curwin *et al.* 2009). A separate high-throughput synthetic dosage lethal screen found that overexpression of *OSH4*, a gene whose function directly antagonizes Sec14 by depleting PI-4-P (Fang *et al.* 1996; LeBlanc and McMaster 2010; Alfaro *et al.* 2011), resulted in decreased viability in the NuA4 mutants *eaf5*Δ and *eaf7*Δ, and lethality in *yng2*Δ (Mitchell *et al.* 2011).

The negative genetic interaction between *sec14-1^ts^* and NuA4 mutants are unexpected as one might anticipate that overproduction of inositol (Opi- phenotype) of the NuA4 mutants would suppress the growth defect in *sec14-1^ts^* cells. This dichotomy provides an elegant means to start dissecting the function of NuA4 within this pathway. Here, we report that deletion or mutation of NuA4 subunits increased the growth defect in *sec14-1^ts^* cells under inositol-depleted conditions. Further investigation through the use of RNA sequencing (RNA-seq) of *sec14-1^ts^eaf1*Δ cells revealed that although transcription of *INO1* was significantly upregulated, the transcription of other ICRE-containing genes was not. This suggests that the role of NuA4 under inositol-depleted conditions extends beyond inositol biosynthesis, most likely through other aspects of phospholipid homeostasis. In fact, we find that NuA4 mutants have impaired lipid droplet levels, which implies defects in the biosynthesis of triacylglycerol (TAG) and steryl esters (SEs). Through genetic and chemical approaches, we determine that the genetic interaction between *sec14-1^ts^* and NuA4 mutants potentially reflects a role for NuA4 in fatty acid biosynthesis. Altogether, our work identifies a role for NuA4 in phospholipid homeostasis through regulation of fatty acid synthesis and lipid droplets.

## Materials and Methods

### Yeast strains, plasmids, and media

All strains used in this study are in the BY4741 (S288C) background and are listed in Table 1. Strains were generated either by standard mating methods or a PCR-mediated gene insertion/deletion technique (Longtine *et al.* 1998). Strains from the Deletion Mutant Array (DMA) collection (GE, catalog no. YSC1053) were confirmed by PCR. A tetracycline/doxycycline inducible promoter (ptet) was used for *CDS1* overexpression, as previously described (Roney *et al.* 2016). All cells were grown in either standard YPD or SC media, as indicated. SC-inositol media was made by mixing 6.7 g/liter of YNB with ammonium sulfate, without inositol (Millipore, catalog no. 4027-412) with 2 g/liter of standard amino acid complete mix. Myo-inositol (Sigma, catalog no. I5125), doxycycline (Sigma, catalog no. D3447), choline chloride (Sigma, catalog no. C7017), or dH_2_O was added to media after autoclaving to the concentration desired. Plates containing cerulenin (Sigma, catalog no. C2389) also contained 1% Brij 58 detergent (Sigma, catalog no. P5884-100G) to help dissolve the drug.

**Table 1:** Strain list

Strain ID	Genotype	Source
YKB1079/BY4741	***MATa*** *his3*Δ*1 leu2*Δ*0 met15*Δ*0 ura3*Δ*0*	Brachmann *et al.* (1998)
YKB3333	***MATa*** *his3*Δ*1 leu2*Δ*0 met15*Δ*0 ura3*Δ*0*	DMA collection (GE)
	*eaf1*Δ::*KANMX*	
YKB3144/CMY505	***MATa*** *his3*Δ*1 leu2*Δ*0 met15*Δ*0 ura3*Δ*0*	Fairn *et al.* (2007)
	*sec14-1^ts^-NATMX*	
YKB3935	***MATa*** *his3*Δ*1 leu2*Δ*0 met15*Δ*0 ura3*Δ*0*	This study
	*sec14-1^ts^-NATMX eaf1*Δ::*KANMX*	
YKB3292	***MATa*** *his3*Δ*1 leu2*Δ*0 met15*Δ*0 ura3*Δ*0*	DMA collection (GE)
	*eaf7*Δ::*KANMX*	
YKB4068	***MATa*** *his3*Δ*1 leu2*Δ*0 met15*Δ*0 ura3*Δ*0*	This study
	*sec14-1^ts^-NATMX eaf7*Δ::*KANMX*	
YKB4236	***MATa*** *his3*Δ*1 leu2*Δ*0 met15*Δ*0 ura3*Δ*0*	This study
	*esa1*Δ::*HIS3 esa1-L254P^ts^-URA3*	
YKB4242	***MATa*** *his3*Δ*1 leu2*Δ*0 met15*Δ*0 ura3*Δ*0*	This study
	*sec14-1^ts^-NATMX esa1*Δ::*HIS3 esa1-L254P^ts^-URA3*	
YKB4325	***MATa*** *his3*Δ*1 leu2*Δ*0 met15*Δ*0 ura3*Δ*0*	This study
	*ade2*Δ::*ptet-CDS1-TAP-LEU2,HIS3*	
YKB4326	***MATa*** *his3*Δ*1 leu2*Δ*0 met15*Δ*0 ura3*Δ*0*	This study
	*ade2*Δ::*ptet-CDS1-TAP-LEU2,HIS3 sec14-1^ts^-NATMX*	
YKB4327	***MATa*** *his3*Δ*1 leu2*Δ*0 met15*Δ*0 ura3*Δ*0*	This study
	*ade2*Δ::*ptet-CDS1-TAP-LEU2,HIS3 eaf1*Δ::*KANMX*	
YKB4328	***MATa*** *his3*Δ*1 leu2*Δ*0 met15*Δ*0 ura3*Δ*0*	This study
	*ade2*Δ::*ptet-CDS1-TAP*::*LEU2,HIS3 sec14-1^ts^-NATMX eaf1*Δ::*KANMX*	
YJP1078	***MATa*** *his3*Δ*1 leu2*Δ*0 met15*Δ*0 ura3*Δ*0*	Gaspar *et al.* (2011)
	*dga1*Δ::*KANMX lro1*Δ::*KANMX are1*Δ::*KANMX are2*Δ::*KANMX*	
YKB4337	***MATa*** *his3*Δ*1 leu2*Δ*0 met15*Δ*0 ura3*Δ*0*	This study
	*sec14-1^ts^-NATMX dga1*Δ::*KANMX lro1*Δ::*KANMX are1*Δ::*KANMX are2*Δ::*KANMX*	
YKB4338	***MATa*** *his3*Δ*1 leu2*Δ*0 met15*Δ*0 ura3*Δ*0*	This study
	*eaf1*Δ::*URA3 dga1*Δ::*KANMX lro1*Δ::*KANMX are1*Δ::*KANMX are2*Δ::*KANMX*	
YKB4339	***MATa*** *his3*Δ*1 leu2*Δ*0 met15*Δ*0 ura3*Δ*0*	This study
	*sec14-1^ts^-NATMX eaf1*Δ*:URA3 dga1*Δ::*KANMX lro1*Δ::*KANMX are1*Δ::*KANMX are2*Δ::*KANMX*	

### Spot assays

Cultures were grown in YPD, SD, or SC –inositol (+75 µM myo-inositol) as indicated at 30° prior to being diluted to an OD_600_ of 0.1, and four times 10-fold serial dilutions (OD_600_ = 0.1, 0.01, 0.001, and 0.0001) were plated and incubated for 2 d at the indicated temperatures. Images of spot assays were taken with the Bio-Rad Chemidoc XRS system under EPI-white light illumination and on the autoexposure setting.

### RNA-seq

The strains *sec14-1^ts^* (CMY505) and *sec14-1^ts^eaf1*Δ (YKB3935) were grown in triplicate in 50 ml of YPD at 30° to midlog growth phase (OD_600_ = 0.5), then shifted to prewarmed YPD media at 33.5° for 2 hr before harvesting, washing with dH_2_O, and flash-freezing on dry ice. Cells were subsequently lysed by resuspending pellets in 100 µl of lyticase reaction solution [1.2 M sorbitol (Sigma, catalog no. S1876); 1 mg/ml lyticase (Sigma, catalog no. SRE0018)] and incubated at 30° for 30 min. RNA was purified using the RNA mini-kit (Ambion, catalog no. 12183018A) following the manufacturer’s yeast extraction protocol. Purified RNA extracts were then twice treated with DNaseI (Promega, catalog no. M6101) for 1 h at 37° each time followed by a phenol/chloroform extraction. After two DNaseI treatments, purified RNA was resuspended in 30 µl of RNase-free water. Three micrograms of purified RNA spiked with 1.5 µg of ERCC RNA control (Ambion, catalog no. 4456740) was depleted of ribosomal RNA (rRNA) using the Ribo-Zero Gold rRNA depletion kit for yeast (Illumina, catalog no. MRZY1306) and resuspended in 10 µl of RNase-free water. Successful depletion of the 18S and 26S rRNA was confirmed using the Agilent Bioanalyzer RNA 6000 Pico quantification kit (Agilent Technologies, catalog no. 5067-1513).

Whole transcriptome RNA-seq libraries were prepared using the Ion Total RNA-Seq Kit V2 (Life Technologies, catalog no. 4479789) and the Ion-Xpress RNA-seq Barcode 1-16 kit (Life Technologies, catalog no. 4475485) according to the manufacturer’s recommendations, using 50 ng of input rRNA depleted total RNA. The size distribution and yield of the final barcoded RNA-seq libraries was assessed using the Agilent Bioanalyzer High Sensitivity DNA quantification kit (Agilent Technologies, catalog no. 5067-4626). All six RNA-seq libraries were multiplexed and sequenced on the Life Technologies Ion Torrent Proton platform using a single Ion Proton Chip.

Resulting reads were aligned against the *S. cerevisiae* S288c genome (version GCF_000146045.2_R64) using the TMAP aligner provided with the Torrent Suite (v5.0.4). Raw sequencing reads have been deposited at the NCBI SRA archive under accession number PRJNA350552. Variation in sample processing during rRNA depletions and library construction was assessed using the ERCC analysis plugin (v5.0.0.0). The number of reads aligning to each annotated transcript was determined using bedtools (v2.17.0) (Quinlan and Hall 2010). Differential expression of transcripts between *sec14-1^ts^* and *sec14-1^ts^eaf1*Δ was determined using DESeq2 (v1.10.1) (Love *et al.* 2014). Transcripts with a fold change of two or more and an adjusted p value ≤0.05 were considered to be differentially expressed. Gene ontology term analysis of enriched biological processes was conducted by using the DAVID bioinformatic online database (https://david.ncifcrf.gov) (Huang da *et al.* 2009). Significantly downregulated or upregulated genes were clustered into functionally related groups using the Gene Functional Classification Tool. The highest ranking Biological Process term was subsequently assigned for each group.

### qRT-PCR

RNA extracts were prepared as above but without rRNA depletion. Purified RNA (2 µg) was reverse transcribed using the High Capacity cDNA Reverse Transcriptase Kit (Applied Biosciences, catalog no. 4368814) and resulting cDNA was diluted 1:10 for qRT-PCR. Transcript levels were measured using the SsoFast EvaGreen Supermix for qPCR (Bio-Rad, catalog no. 172-5201) with the Bio-Rad MiniOpticon Real-Time PCR system and CFX manager software. Primers used for qRT-PCR are as follows: (1) *INO1* forward (Forw) primer: 5′-TCTCTGTTGCCCATGGTTAG-3′ and reverse (Rev) primer: 5′-CTTCAAGCGTTGTTGCAGAT-3′; (2) *FAS1* Forw: 5′-TGAAGGTGTTCCATCTCCAA-3′ and Rev: 5′-CGACTAGATTCTT-CGCACCA-3′; *FAS2* Forw: 5′-GCCAGAACAAGATGGGAAAT-3′ and Rev: 5′-TGTATGGACGACCCTTCAAA-3′; *CDS1* Forw: 5′-GCCATGGTGGTATCACAGAC-3′ and Rev: 5′-TCTGCTTGTCGTTCAGGTTC-3′; *YEH1* Forw: 5′-TTCTCAGGGCACTACACAGG-3′ and Rev: 5′-AAGGGACCAGGATACACTGC-3′; *BCP1* Forw: 5′-TGATTAATATGCCACCGGAA-3′ and Rev: 5′-ATGTTTGTCATCGCCAAGTG-3′; *TDH3* Forw: 5′-CTGTCAAGTTGAACAAGGAAACCAC-3′ and Rev: 5′-CAACGTGTTCAACCAAGTCGACAA-3′; *ACT1* Forw: 5′-GCCTTCTACGTTTCCATCCA-3′ and Rev: 5′-CGTAAATTGGAACGACGTGA-3′. A standard curve was constructed using a five times fivefold serial dilution series of pooled cDNA sample from the original individual reverse transcription reactions starting at a 1:5 dilution. The geometric averaging of two internal reference genes, *TDH3* and *ACT1*, was used for normalization of relative expression levels (Pfaffl 2001) using the Bio-Rad CFX Manager software. Three biological and two technical replicates were used for each sample. Statistical analyses were completed using a two-tailed unpaired *t*-test (p ≤ 0.05) with the GraphPad Prism software.

### Immunoblotting

Samples were grown in SC media at 30° until midlog growth phase (OD_600_ = 0.5–0.6), then shifted to SC media with indicated amounts of doxycycline for 4 hr, before being harvested by centrifugation, washed in water, aliquoted in 1.5-ml Eppendorf tubes, and flash frozen in liquid nitrogen. Pellets were resuspended in 30% TCA lysis buffer and lysed mechanically by vortex with glass beads. Cell debris and glass beads were removed by centrifugation. Whole cell extract (WCE) was diluted in Laemmli loading dye and stored at −80°. The prepared samples were boiled at 95° for 10 min prior to separation by SDS-PAGE on a linear SDS-polyacrylamide gel (7.5%). Western blotting was performed using a semidry transfer apparatus from Bio-Rad (Trans-Blot SD Semi Dry Electrophoretic Transfer Cell; catalog no. 170-3940). Blocking, primary, and secondary incubations were performed with 5% milk in TBS-T (0.1% Tween 20; VWR, catalog no. CA95017-122L). Primary incubations were carried out at 4° overnight with rabbit α-TAP antibodies (1:10,000 dilution; Thermo Fisher, catalog no. PICAB1001) and secondary incubations at room temperature for 1 hr with peroxidase-conjugated goat α-rabbit IgG (1:10,000 dilution; Chemicon, catalog no. AP307P). Chemiluminescence was detected using Immobilon Western Chemiluminescent HRP substrate (Millipore, catalog no. WBKLS0500) and developed on a ChemiDoc XRS system (Bio-Rad, catalog no. 170-8070).

### Lipid droplet

Cultures were grown in SC-inositol (+75 µM myo-inositol) media at 30° to midlog growth phase (OD_600_ = 0.5), washed in dH_2_O, and transferred to SC-inositol media supplemented with or without 75 µM of myo-inositol and grown for 2 hr at 30°. Cells were subsequently incubated with 10 µM BODIPY 493/503 (Life Technologies, catalog no. D3922) for 10 min at room temperature in their residing media and washed with dH_2_O. Cells were pelleted, resuspended in SC-inositol media (+ or − inositol), and loaded onto prewashed microscope slide with coverslips. Images were taken using the CellVoyager CV1000 disk confocal microscope (Yokogawa Electric Corporation, Musashino Tokyo, Japan). Brightfield (20% intensity, 100-ms exposure time, 100% gain) and BODIPY fluorescence, using FITC filters (25% intensity, 50-ms exposure time, 20% gain), were taken across multiple fields of view to capture at least 100 cells on the 100× oil immersion objective. Images for each field of view were taken at 0.2-µm steps for a total of 30 images. Image analysis was done using a custom MATLAB script. Bright field images were first thresholded using an adaptive thresholding algorithm. The resulting thresholded images were segmented using our MATLAB script that incorporated previously published code (Ricicova *et al.* 2013), as well as our own code that makes use of functions found in the MATLAB Image Processing Toolbox. The cell outlines obtained through segmentation were used to quantify the area (in pixels) and total fluorescence from the fluorescence images. Fluorescence was quantified using the middle *z*-stack for each cell. Fluorescence density was calculated by dividing total fluorescence by area. A total of three biological replicates with >100 cells were analyzed for each sample. Mean fluorescence for each sample are converted relatively to wild-type samples in inositol-supplemented conditions. Statistical analyses were performed by one-way ANOVA with the Tukey’s multiple comparisons test, using the GraphPad Prism software.

### Data availability

Strains and code are available upon request. Supplemental Material, File S1 contains the complete RNA-seq data set and gene functional classification analysis. Raw sequencing reads have been deposited at the NCBI SRA archive under accession number PRJNA350552.

## Results

### NuA4 mutants exacerbate the growth defects of sec14-1^ts^ under inositol-depleted conditions

**Figure 2:**
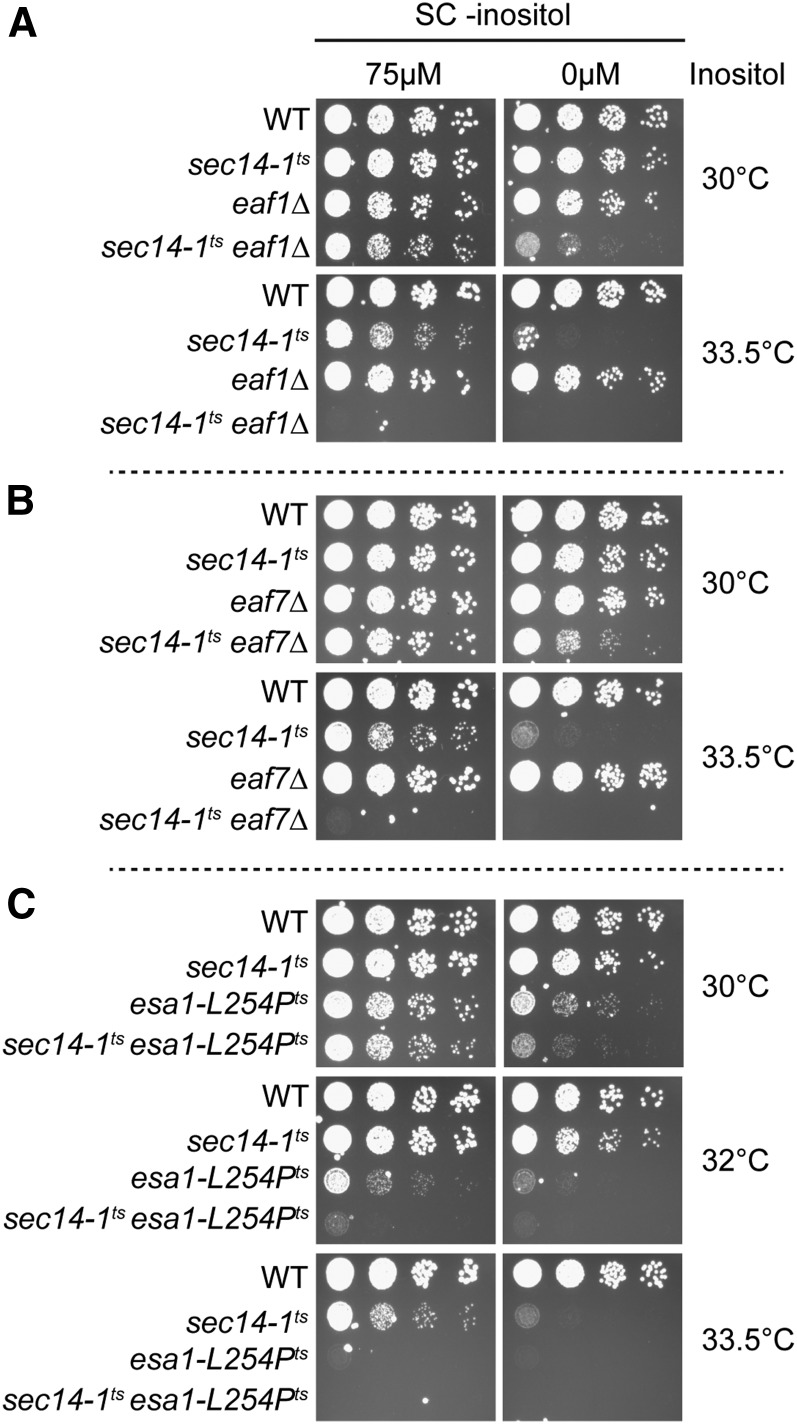
NuA4 mutants exacerbate the growth defects of *sec14-1^ts^* on inositol-depleted media. NuA4 mutants (A) *eaf1*Δ, (B) *eaf7*Δ, and (C) *esa1-L254P^ts^* increase the growth defects of the *SEC14* temperature-sensitive mutant, *sec14-1^ts^*, under inositol-depleted conditions. Wild-type (YKB1079), *sec14-1^ts^* (YKB3144), *eaf1*Δ (YKB3333), *sec14-1^ts^eaf1*Δ (YKB3935), *eaf7∆* (YKB3292), *sec14-1^ts^eaf7∆* (YKB4068), *esa1-L254P^ts^* (YKB4236), and *sec14-1^ts^esa1-L254P^ts^* (YKB4242) cultures were grown to midlog phase in SC-inositol (+75 µM myo-inositol) prior to being diluted to an OD_600_ of 0.1, and four times 10-fold serial dilutions were spotted on inositol-supplemented (75 µM myo-inositol) SC media or inositol-depleted SC media. Plates were incubated for 2 d at 30, 32, or 33.5° and images are representative of three biological replicates.

The growth defects of *sec14-1^ts^* on inositol-depleted media can be suppressed by mutants which exhibit an overproduction of inositol phenotype (Opi-) due to the derepression and transcription of the rate-limiting biosynthetic enzyme within this pathway, *INO1* (Patton-Vogt *et al.* 1997; Chang *et al.* 2002). As NuA4 mutants exhibit a derepression of *INO1* and excessive inositol production, or Opi- phenotype (Hancock *et al.* 2006; Salas-Santiago and Lopes 2014), we anticipated that NuA4 mutants would suppress the growth defect in *sec14-1^ts^* under inositol-depleted conditions. To test this hypothesis, NuA4 mutants *eaf1*Δ and *eaf7*Δ, and the temperature sensitive *ESA1* mutant, *esa1-L254P^ts^* (Clarke *et al.* 1999), were crossed with *sec14-1^ts^* and growth was assessed under both inositol-supplemented and -depleted conditions (Figure 2). As expected, *sec14-1^ts^eaf7*Δ cells display synthetic sickness upon increased temperature in inositol-supplemented media (Curwin *et al.* 2009), and similar interactions were seen for the double mutants of *sec14-1^ts^* with *eaf1*Δ and *esa1-L254P^ts^*. As was also expected, *sec14-1^ts^* cells exhibited a growth defect in inositol-depleted media at the semipermissive temperature of 33.5° (Patton-Vogt *et al.* 1997) (Figure 2A). While the *eaf1*Δ and *eaf7*Δ single mutants grew similarly between both conditions (Figure 2, A and B), we were surprised to see that *esa1-L254P^ts^* cells displayed growth defects in inositol-depleted media even at 30° (Figure 2C). In combination, the double mutants *sec14-1^ts^eaf1*Δ, *sec14-1^ts^eaf7*Δ, and *sec14-1^ts^esa1-L254P^ts^* all showed a synthetic growth defect at 30° in inositol-depleted media compared to *sec14-1^ts^* (Figure 2). These results suggest that, although *eaf1*Δ and *eaf7*Δ do not display detectable growth defects under inositol-depleted conditions like *esa1-L254P^ts^*, these mutants do in fact exacerbate the inositol-depleted growth defects for *sec14-1^ts^* under normally permissive temperatures. As NuA4 mutants derepress *INO1* transcription and secrete excessive amounts of inositol (Hancock *et al.* 2006; Salas-Santiago and Lopes 2014), the root cause of the growth defect under inositol-depleted conditions of *sec14-1^ts^eaf7*Δ, *sec14-1^ts^eaf1*Δ, and *sec14-1^ts^esa1-L254P*^ts^ cells appears independent of inositol production.

### ICRE-regulated phospholipid genes are not fully derepressed in an EAF1 mutant background

**Figure 3:**
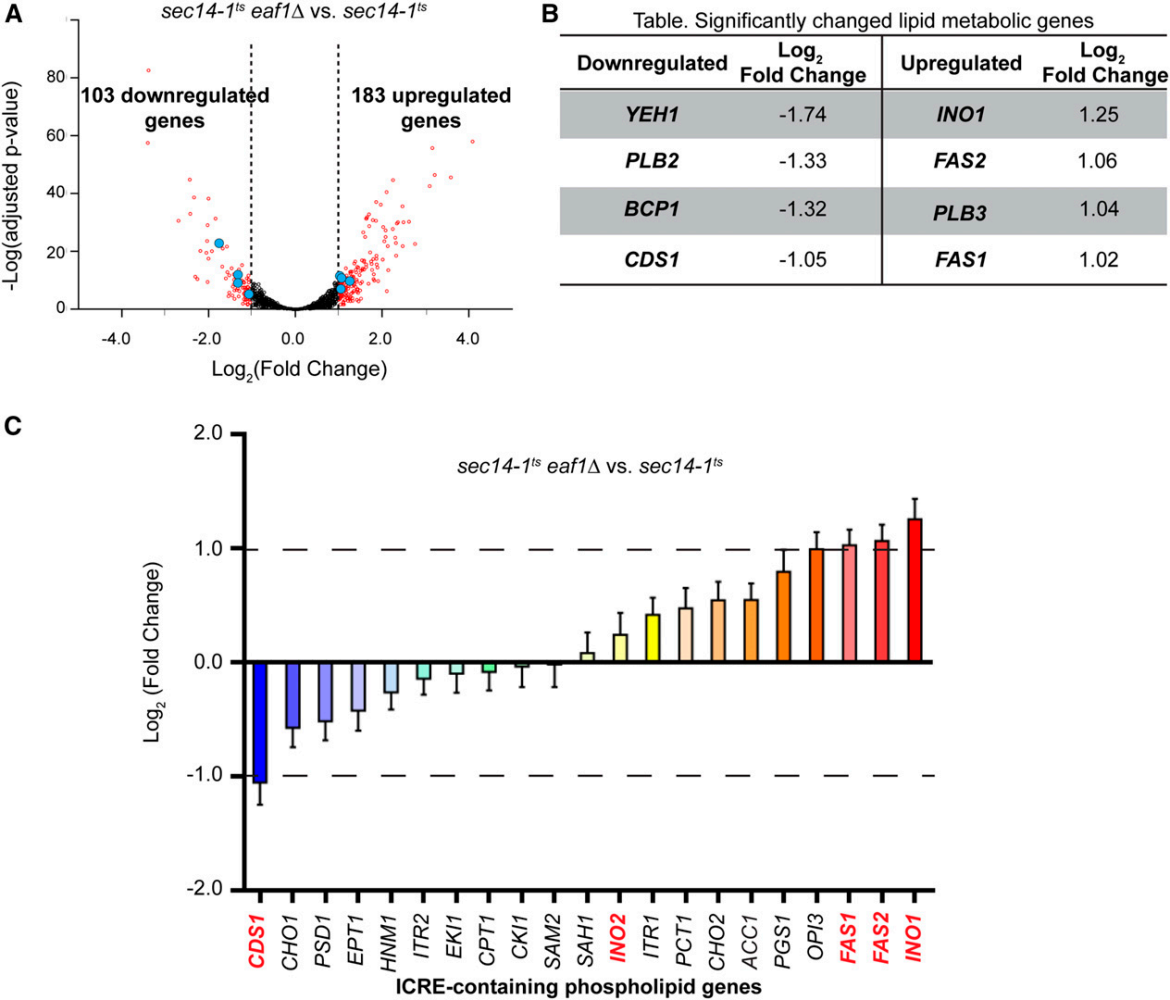
Transcriptional profiling of *sec14-1^ts^eaf1*Δ reveals that most ICRE-regulated phospholipid genes are not significantly derepressed. (A) Volcano plot for differentially expressed genes, *sec14-1^ts^eaf1*Δ (YKB3935) *vs.* *sec14-1^ts^* (YKB3144), from RNA sequencing experiments. Differential expression levels of aligned sequences were calculated using significant thresholds set at log_2_ fold change over two and FDR adjusted p-value ≤ 0.05. Significant differentially expressed genes are in red. Blue shows the significantly changed lipid metabolic genes described in (B). (B) Table listing significantly downregulated and upregulated lipid metabolic genes. (C) Relative expression levels of ICRE-containing phospholipid genes between *sec14-1^ts^eaf1*Δ and *sec14-1^ts^*. Genes are ordered from lowest to highest fold-change expression. Genes in red are discussed further in the text.

We next sought to determine if ICRE-regulated or phospholipid genes were derepressed in a NuA4 mutant under conditions where Sec14 is inactivated, and if so, would dysregulation of these genes explain the genetic interactions between *sec14-1^ts^* and NuA4 mutants. Using next-generation RNA-seq, we compared mRNA gene expression between *sec14-1^ts^* and *sec14-1^ts^eaf1*Δ. RNA samples from three biological replicates were extracted from midlog phase cells that were shifted to semipermissive temperatures (33.5°) for 2 hr to briefly inactivate *sec14-1^ts^*. In summary, >8 million reads were obtained for each sample, identifying at least 89% of the annotated open reading frames in the *Saccharomyces* genome database (Cherry *et al.* 2012; Engel *et al.* 2014). Analysis of the ERCC spike-in standard revealed that all samples had an *R*^2^ correlation ranging from 0.92 to 0.95 (Figure S1A in File S2). These results indicate our rRNA depletion and library construction was consistent and unbiased across samples. Principal component analysis determined that the majority of the variance between samples is attributable to the differences between the two different strains and not their biological replicates (Figure S1B in File S2). Only a subset of genes was found to be significantly downregulated (103 genes, 1.84%) or upregulated (183 genes, 3.11%) in *sec14-1^ts^eaf1*Δ compared to *sec14-1^ts^* by a fold change of two or more and FDR adjusted p-value ≤ 0.05 (Figure 3A and File S1).

Deletion of *EAF1* in the *sec14-1^ts^* background resulted in the decreased expression of the purine biosynthesis genes (*ADE1*, *ADE12*, *ADE17*) phosphate metabolic genes (*PHO3*, *PHO5*, *PHO11*, *PHO12*) and a large cluster of genes associated with ribosomal biogenesis (File S1), which is in agreement with previously published transcriptome studies on NuA4 mutants (Reid *et al.* 2000; Krogan *et al.* 2004; Nourani *et al.* 2004; Lindstrom *et al.* 2006; Cheng *et al.* 2015). Upregulated genes common to previous *eaf1*Δ transcriptome studies and our *sec14-1^ts^eaf1*Δ transcriptome included the stress response genes (*DDR2*, *HSP12*, *HSP26*) (Lindstrom *et al.* 2006; Cheng *et al.* 2015). Together, these results indicate that deletion of *EAF1* in the *sec14-1^ts^* background impacts the transcriptome similar to what has been previously reported for NuA4 mutants in the wild-type background. However, our main purpose was to assess if ICRE-containing genes or genes implicated in phospholipid metabolism were differentially regulated by NuA4 in the *sec14-1^ts^* background. 

If NuA4 is regulating transcription of ICRE-containing genes, one would anticipate that other ICRE-containing phospholipid metabolic genes would be similarly derepressed as *INO1* (Figure 3C); however, this was not the case. While the expression of the fatty acid synthases *FAS1* and *FAS2* (Kuziora *et al.* 1983) were significantly upregulated in *sec14-1^ts^eaf1*Δ cells, the expression of phosphatidate cytidylyltransferase (CDP-diglyceride synthetase) *CDS1* (Shen *et al.* 1996; Shen and Dowhan 1997) was downregulated (Figure 3C). Indeed, the expression of the majority of ICRE-containing phospholipid genes did not display significant changes in gene expression in our transcriptome profile, including the transcription factor *INO2*, whose expression remained close to basal level (Figure 3C). Derepression of *INO1* expression is normally preceded by increased levels of *INO2* which dimerizes with *INO4* and binds to ICRE gene promoters to promote transcription (Ambroziak and Henry 1994; Ashburner and Lopes 1995). Our transcriptome analysis provides evidence of *INO1* derepression occurring independently of *INO2* derepression, suggesting that the transcriptional derepression of *INO1* in *sec14-1^ts^eaf1*Δ is not characteristic of the typical Opi- transcriptional response.

Extending our search beyond just ICRE-regulated genes, we were surprised to find that only eight genes involved in phospholipid metabolism displayed significant transcriptional change between *sec14-1^ts^* and *sec14-1^ts^eaf1*Δ (Figure 3B). In addition to *CDS1*, downregulated genes included the SE hydrolase *YEH1* (Koffel *et al.* 2005), the phospholipase B *PLB2* (Fyrst *et al.* 1999), and *BCP1*, which encodes a protein implicated in phosphatidylinositol-4,5-bisphosphate synthesis (Audhya and Emr 2003). Upregulated genes included the inositol-3-phosphate synthase *INO1*, the phospholipase B *PLB3* (Merkel *et al.* 1999), and *FAS1* and *FAS2*. As recent evidence suggests that the contribution of phospholipase B enzymes toward phospholipid turnover *in vivo* is negligible (Mora *et al.* 2012), we focused our attention on the remaining six genes. For each of the lipid metabolic genes, the changes in expression detected by next-generation sequencing analysis were confirmed through qRT-PCR (Figure S2 in File S2).

The misregulation of a handful of phospholipid metabolic genes, most notably that of *CDS1*, suggests that perturbation of phospholipid homeostasis may contribute to the growth defects displayed in *sec14-1^ts^eaf1*Δ under inositol-depleted conditions. Given the essential role of *CDS1*, we predicted that its downregulation in *sec14-1^ts^eaf1*Δ may be contributing to this negative genetic interaction. However, we were surprised to discover that the overexpression of *CDS1* in *sec14-1^ts^eaf1*Δ could not rescue the growth defects under inositol-depleted conditions (Figure S3 in File S2). Therefore, downregulation of *CDS1* by itself is not the cause of the growth defects in *sec14-1^ts^eaf1*Δ.

### Lipid droplet dynamics is impaired in eaf1**Δ** cells

**Figure 4:**
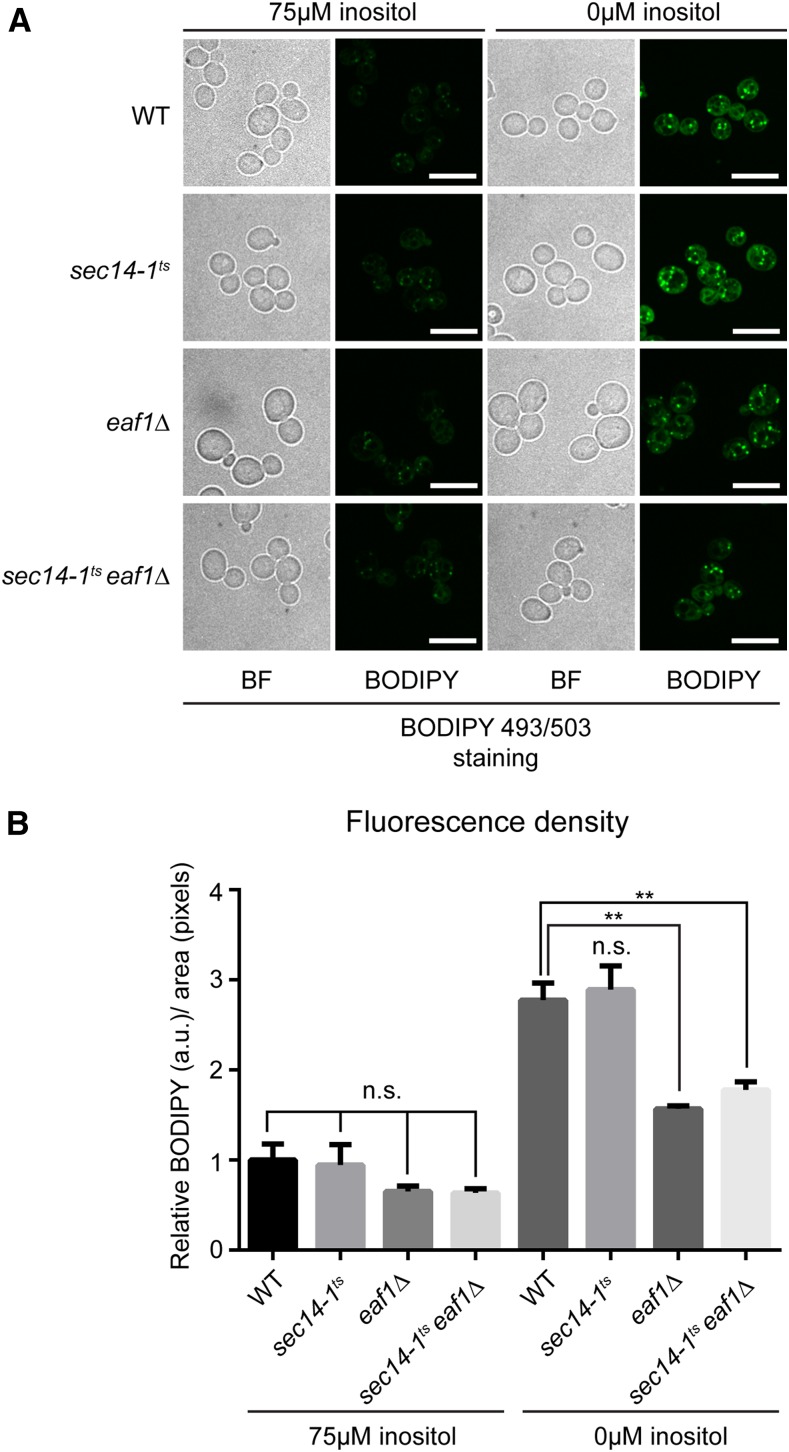
Lipid droplet dynamics is impaired in *EAF1* deletion mutants. (A) Wild-type (YKB1079), *sec14-1^ts^* (YKB3144), *eaf1*Δ (YKB3333), and *sec14-1^ts^eaf1*Δ (YKB3935) cultures were grown to midlog phase in SC media at 30° before shifting to inositol-supplemented (75 µM myo-inositol) SC media or inositol-depleted SC media for 2 hr at 30° prior to being stained with 10 µM BODIPY 493/503 for 10 min before imaging. Images shown are midfield view of representative cells for each sample (scale bar, 10 µm). (B) Graphic display of the mean relative fluorescence density for each strain and condition. Cells were first segmented from brightfield images using a custom MATLAB script then fluorescence was measured for each cell using the outlines (see *Materials and Methods* for details). Mean fluorescence density was measured from 100 cells across three biological replicates relative to wild-type fluorescence in inositol-supplemented media (+SEM). Statistical analysis was performed by one-way ANOVA with Tukey’s multiple comparisons test: **p < 0.05; n.s., not significant.

We next wanted to determine which, if any, lipid metabolic pathways were affected in *eaf1*Δ cells that might explain the genetic interaction between *sec14-1^ts^* and *eaf1*Δ. We opted to explore if the growth defect in *sec14-1^ts^eaf1*Δ under inositol-depleted conditions is potentially due to defects in TAG and SE biosynthesis for two reasons. First, we found altered expression of *CDS1*, *FAS1*, and *FAS2*, which all encode proteins implicated in neutral lipid synthesis (reviewed in Eisenberg and Buttner 2014; Fernandez-Murray and McMaster 2016) (Figure 1). Second, under inositol-depleted conditions, cells require inositol as well as the ability to synthesize both TAG and SE (both neutral lipids) for survival (Gaspar *et al.* 2011). TAG and SE biosynthesis can be completely abolished in yeast by deleting *DGA1*, *LRO1*, *ARE1*, and *ARE2*, genes encoding the proteins responsible for all neutral lipid biosynthesis in yeast (Oelkers *et al.* 2002; Sandager *et al.* 2002). Intriguingly, like *sec14-1^ts^eaf1*Δ strains, a yeast strain in which all TAG and SE biosynthesis is abolished (*dga1*Δ*lro1*Δ*are1*Δ*are2*Δ) is not able to grow in the absence of inositol at higher temperatures despite *INO1* expression (Gaspar *et al.* 2011). The formation of lipid droplets is an established proxy for TAG and SE biosynthesis (Wang 2015); therefore, we examined lipid droplet formation under inositol-supplemented and -depleted conditions in the wild-type, *sec14-1^ts^*, *eaf1*Δ, and *sec14-1^ts^eaf1*Δ strains at 30° using a fluorescent lipid droplet marker. As expected, upon inositol depletion, wild-type cells display a nearly threefold increase in fluorescence of the lipid droplet marker, whereas there is no increase in *dga1*Δ*lro1*Δ*are1*Δ*are2*Δ cells where TAG and SE biosynthesis is abolished (Figure 4 and Figure S4 in File S2) (Gaspar *et al.* 2011). Much like wild-type cells, upon inositol depletion, *sec14-1^ts^* cells emitted a nearly threefold increase in fluorescence density compared to inositol-supplemented conditions (Figure 4, A and B). In contrast, deletion of *EAF1* in either the wild-type or *sec14-1^ts^* background resulted in a reduction in the fluorescence intensity of lipid droplets, which suggests that some aspect of lipid droplet dynamics is significantly impaired in *eaf1*Δ cells. Furthermore, although the reduction of fluorescence intensity of lipid droplets was not as striking in *eaf7*Δ and *sec14-1^ts^eaf7*Δ cells, it was clearly reduced in *esa1-L254P^ts^* and *sec14-1^ts^esa1-L254P^ts^* cells. As both *eaf1*Δ and *esa1-L254P^ts^* cells display reduced KAT activity, this suggests that the catalytic activity of the NuA4 complex is involved in the regulation of lipid droplet and potentially neutral lipid biosynthesis (Figure S5 in File S2). If the defect in lipid droplets seen in NuA4 mutant cells is a reflection of neutral lipid biogenesis, this potentially could explain the increased growth defects of *sec14-1^ts^eaf1*Δ, *sec14-1^ts^eaf7*Δ, and *sec14-1^ts^esa1-L254P^ts^* cells under inositol-depleted conditions. 

### Inhibition of de novo fatty acid biosynthesis has a detrimental effect on sec14-1^ts^ growth

**Figure 5:**
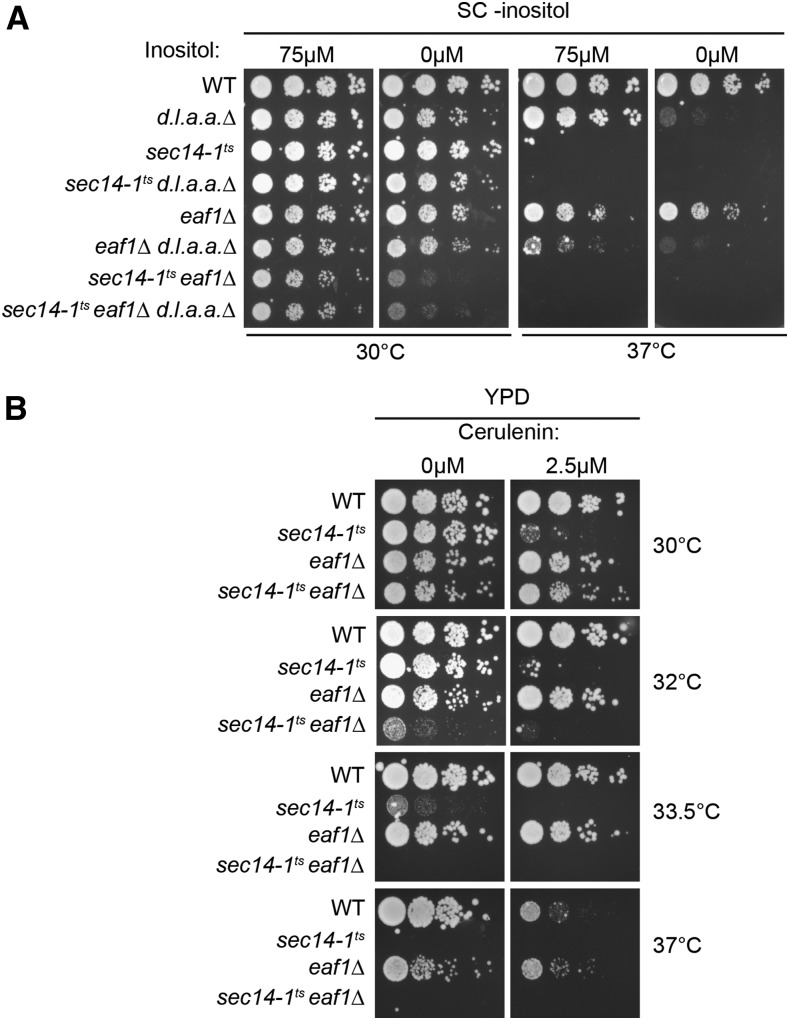
Inhibition of fatty acid biosynthesis causes a synthetic growth defect in *sec14-1*^ts^. (A) Genetic inhibition of triacylglycerol and steryl ester biosynthesis does not affect the growth of *sec14-1^ts^*. Wild-type (YKB1079), *dga1*Δ*lro1*Δ*are1*Δ*are2*Δ (*d.l.a.a*.Δ; YKB4336), *sec14-1^ts^* (YKB3144), *sec14-1^ts^d.l.a.a*.Δ (YKB4337), *eaf1*Δ (YKB3333), *eaf1*Δ*d.l.a.a*.Δ (YKB4338), *sec14-1^ts^eaf1*Δ (YKB3935), and *sec14-1^ts^eaf1*Δ *d.l.a.a*.Δ (YKB4339) cultures were grown in YPD at 30° prior to being diluted to an OD_600_ of 0.1, and four times 10-fold serial dilutions were plated on inositol-supplemented (75 µM myo-inositol) SC media or inositol-depleted SC media and incubated for 2 d at 30°. (B) Inhibition of fatty acid biosynthesis by cerulenin treatment causes synthetic growth defect with *sec14-1^ts^*. Wild-type (YKB1079), *sec14-1^ts^* (YKB3144), *eaf1*Δ (YKB3333), and *sec14-1^ts^eaf1*Δ (YKB3935) cultures were grown in YPD at 30° prior to being diluted to an OD_600_ of 0.1, and four times 10-fold serial dilutions were plated on YPD with indicated amounts of cerulenin for 2 d at 30, 32, 33.5, and 37°. Images are representative of three biological replicates.

To investigate if the genetic interaction between *sec14-1^ts^* and *eaf1*Δ was due to a role of Eaf1 (and presumably NuA4) in neutral lipid biosynthesis, we examined the growth of *sec14-1^ts^* strains when both TAG and SE biosynthesis are eliminated (Figure 5A). As previously shown, *dga1*Δ*lro1*Δ*are1*Δ*are2*Δ (*d.l.a.a*.Δ in Figure 5) cells do not display defects on inositol-supplemented media, but become inositol auxotrophs at 37° (Gaspar *et al.* 2011) (Figure 5A). If the genetic interaction between *sec14-1*^ts^ and *eaf1*Δ was due to decreased neutral lipid biosynthesis caused by deletion of *EAF1*, we would predict that *sec14-1^ts^dga1*Δ*lro1*Δ*are1*Δ*are2*Δ cells would display similar growth defects on inositol-depleted media as *sec14-1^ts^eaf1*Δ. However, in inositol-supplemented or -depleted conditions at 30°, *dga1*Δ*lro1*Δ*are1*Δ*are2*Δ combined with *sec14-1^ts^* does not impair growth. Further, the addition of *dga1*Δ*lro1*Δ*are1*Δ*are2*Δ does not increase the growth defects of *sec14-1^ts^eaf1*Δ cells at 30°. Indeed, we detect a negative genetic interaction between *eaf1*Δ and *dga1*Δ*lro1*Δ*are1*Δ*are2*Δ on inositol-supplemented media at 37°, suggesting that Eaf1, and presumably NuA4, function in separate pathways. Altogether, this suggests the growth defect in *sec14-1^ts^eaf1*Δ under inositol-depleted conditions or at higher temperatures is not the result of *eaf1*Δ mutant defects in neutral lipid biosynthesis.

Though a reduction in neutral lipid biosynthesis does not explain the inositol-depleted growth defects of *sec14-1^ts^eaf1*Δ cells, the reduction of lipid droplet staining in NuA4 mutant cells suggests a potential defect(s) within this pathway (Figure 4 and Figure S5 in File S2). Therefore, we examined whether the *de novo* biosynthesis of fatty acids (Figure 1), a requisite precursor for neutral lipid biosynthesis, was involved in the negative genetic interaction between *sec14-1^ts^* and *eaf1*Δ. For this purpose, we used cerulenin, a fatty acid analog that inhibits the enzymatic activity of fatty acid synthases *FAS1* and *FAS2* (Kawaguchi *et al.* 1979, 1982), to determine the effect of inhibiting fatty acid biosynthesis on *sec14-1^ts^* growth. We found that while the growth of wild-type and *eaf1*Δ cells were not impacted by 2.5 µM cerulenin treatment at 30°, growth of *sec14-1^ts^* cells were hypersensitive to cerulenin treatment, suggesting that fatty acid biosynthesis is especially critical for the survival of this strain (Figure 5B). Interestingly, the cerulenin sensitivity of *sec14-1^ts^* cells at 30° was rescued by deletion of *EAF1*. However, at 32° and 33.5°, cerulenin treatment affected *sec14-1*^ts^ growth to a similar extent as *sec14-1^ts^eaf1*Δ in the absence of cerulenin treatment, and the deletion of *EAF1* in the *sec14-1^t^*^s^ background did not increase sensitivity to cerulenin treatment at 32°. Moreover, at 37°, *eaf1*Δ cells normally display a growth defect compared to wild-type cells; however, upon cerulenin treatment, wild-type cells lose their growth advantage, with both wild-type and *eaf1*Δ cells displaying comparable growth. A similar buffering effect was also seen with *eaf7*Δ and *esa1-L254P^ts^* mutants upon cerulenin treatment (Figure S6 in File S2). This suggests a role for NuA4 within the fatty acid biosynthesis pathway that helps buffer the inhibition of the fatty acid synthases. Taken together, this work suggests that the *eaf1*Δ mutant negative genetic interaction with *sec14-1*^ts^ and the decreased lipid droplet staining in *eaf1*Δ originate from defects within the fatty acid biosynthesis pathway.

## Discussion

Here we set out to begin to dissect the biological role for NuA4 in phospholipid homeostasis. Surprisingly, we found that NuA4’s role in inositol auxotrophy is not simply a reflection of regulation of ICRE-regulated phospholipid genes. Rather, through dissection of the genetic interaction between *sec14-1^ts^* and *eaf1*Δ, our work provides evidence for a role of NuA4 in contributing to the regulation of neutral lipid and fatty acid biosynthesis pathway.

### NuA4 mutants increase the inositol auxotrophy of sec14-1^ts^ cells despite INO1 derepression

NuA4 mutants are known to exhibit the Opi- phenotype, which is the overproduction of inositol caused by the derepression and transcription of *INO1* (Hancock *et al.* 2006; Salas-Santiago and Lopes 2014). As deletion mutants of the CDP-choline pathway that exhibit an Opi- phenotype suppress the growth defect in the *sec14-1^ts^* mutant under inositol-depleted conditions (Patton-Vogt *et al.* 1997; Chang *et al.* 2002), we initially hypothesized that the introduction of NuA4 mutants into a *sec14-1^ts^* background would rescue growth on inositol-depleted media. Instead, we found that the combination of *sec14-1^ts^* and NuA4 mutants resulted in an increased growth defect under inositol-depleted conditions despite *INO1* derepression (Figure 2, Figure 3, and Figure S2A in File S2). However, comparative to *opi1*Δ cells that are considered to have a strong Opi- phenotype, NuA4 mutants display only an intermediate Opi- phenotype, as determined by the extent of the depression of an *INO1-CYC-lacZ* reporter gene (Hancock *et al.* 2006). Similarly, our transcriptome analysis indicated only a twofold derepression of *INO1* expression (Figure 3), which differs considerably from the 56.7-fold increase in an *opi1*Δ background (Santiago and Mamoun 2003). Despite this fact, we did not expect the growth defect in *sec14-1^ts^* under inositol-depleted conditions to worsen in combination with NuA4 mutants. This suggests that survival under inositol-depleted conditions requires more than just *INO1* induction and inositol production. Our transcriptional data showed that partial derepression and transcription of *INO1* was occurring in *sec14-1^ts^eaf1*Δ, but derepression was not extended to all ICRE-regulated phospholipid genes. The main transcriptional activator of ICRE-regulated genes, *INO2*, which is normally induced under similar conditions as *INO1*, remained at basal levels of transcription in *sec14-1^ts^eaf1*Δ cells (Figure 3). This suggests that the induction of *INO1* in *sec14-1^ts^eaf1*Δ cells and other NuA4 mutants (Hancock *et al.* 2006; Salas-Santiago and Lopes 2014) may not be dependent on *INO2*, potentially explaining why most ICRE-regulated genes are not induced in *sec14-1^ts^eaf1*Δ cells.

One possible mechanism for the induction of *INO1* expression in *sec14-1^ts^eaf1*Δ cells could be through the downregulation of *CDS1* expression (Figure 3 and Figure S2D in File S2). It has been shown that lowering expression of the essential gene *CDS1* causes an Opi- phenotype and induction of *INO1* expression independent of *INO2* derepression, much like NuA4 mutants (Shen and Dowhan 1996, 1997; Salas-Santiago and Lopes 2014). Therefore, it is possible that the Opi- phenotype previously reported for NuA4 mutants is caused by the downregulation of *CDS1* expression. However, reduction of *CDS1* causes an increase in both PA and TAG levels, resulting in supersized lipid droplets (Fei *et al.* 2011), which is not seen in *eaf1*Δ cells (Figure 4). Furthermore, we have shown that overexpressing *CDS1* does not rescue the growth phenotype of our *eaf1*Δ strain (Figure S3 in File S2). One intriguing possibility is the Opi- phenotype of NuA4 mutants is an indirect consequence of the cell trying to compensate for defects in TAG and SE biosynthesis, resulting in the reduction of *CDS1*. Indeed, it remains to be determined if NuA4-dependent acetylation of histones is directly regulating the transcription of the eight lipid metabolism genes identified in our screen, or if the changes in gene expression reflect a transcriptional compensation mechanism independent of NuA4’s nuclear activity.

### Is NuA4 regulating lipid droplet dynamics through the fatty acid biosynthesis pathway?

A decrease in lipid droplet staining despite the derepression of *INO1* expression is also a characteristic of the quadruple mutant *dga1*Δ*lro1*Δ*are1*Δ*are2*Δ, which is unable to synthesize neutral lipids (Figure S4 in File S2) (Mullner and Daum 2004; Gaspar *et al.* 2011). Further, like our *esa1-L254P^ts^* mutant cells (Figure 2C), *dga1*Δ*lro1*Δ*are1*Δ*are2*Δ cells are sensitive to inositol-depleted conditions at higher temperatures (Figure 5A) (Gaspar *et al.* 2011). However, the loss of neutral lipid biosynthesis did not account for the negative genetic interaction between NuA4 and Sec14 mutants, nor did it account for the increased growth of *sec14-1^ts^eaf1*Δ on inositol-depleted media at 30° (Figure 5A).

An alternative explanation could be that defective lipid droplet formation in *eaf1*Δ and other NuA4 mutants is caused by a lack of fatty acid substrate. We demonstrated that the inhibition of the fatty acid biosynthesis pathway by cerulenin dramatically increased the growth defect in *sec14-1^ts^* (Figure 5B and Figure S6 in File S2). As the deleterious effect of cerulenin treatment on *sec14-1^ts^* is only mildly additive to the growth defects incurred by NuA4 mutants at 32°, it would suggest that fatty acid biosynthesis may already be compromised in NuA4 mutants at higher temperatures. Indeed, this could account for the limited impact of cerulenin treatment on *eaf1*Δ cells compared to wild-type cells at 37°. The suppressive effect of *sec14-1^ts^eaf1*Δ, *sec14-1^ts^eaf7*Δ, and *sec14-1^ts^esa1-L254P^ts^* on cerulenin treatment compared to *sec14-1^ts^* at 30° remains unresolved. Potentially, increased expression of both *FAS1* and *FAS2* in *sec14-1^ts^eaf1*Δ (Figure 3 and Figure S2, B and C in File S2), and presumably other NuA4 mutants, may be able to buffer the effects of cerulenin at 30°; however, at higher temperatures, increased *FAS1/2* expression may not be able to buffer the defects within this pathway manifested by deletion of *EAF1*. The fact remains that the effect of inhibiting fatty acid synthases in *sec14-1^ts^* cells is largely nonadditive to the effect of NuA4 mutants, which suggests an epistatic interaction between NuA4 and *FAS1/FAS2*.

Our proposed theory that NuA4 positively regulates the fatty acid biosynthesis pathway accounts for most of the phenotypes reported in this paper. Inhibition of the fatty acid biosynthesis pathway negatively affects neutral lipid biosynthesis and lipid droplet formation as they are the principal substrates in these pathways (Henry *et al.* 2012; Wang 2015). Similar to NuA4 mutants, downregulation of the fatty acid biosynthesis pathway leads to the derepression and transcription of *INO1* (Shirra *et al.* 2001). Whether the inhibition of the fatty acid biosynthesis pathway is the primary cause behind *INO1* transcriptional activation in NuA4 mutants remains to be confirmed. Conversely, the opposite effect is observed when this pathway is activated. Overexpression of genes responsible for fatty acid biosynthesis represses the transcription of *INO1* (Shirra *et al.* 2001; Feddersen *et al.* 2007) and increases fatty acid biosynthesis, causing a dramatic accumulation of lipid droplets (Hofbauer *et al.* 2014).

How is NuA4 regulating the fatty acid biosynthesis pathway? Our work suggests that NuA4 is a positive regulator of the fatty acid biosynthesis pathway. Based on this theory, one potential mechanism is through NuA4-dependent transcriptional regulation of fatty acid biosynthesis genes. However, transcriptome analysis on NuA4 mutant subunits have not identified significant decreases in fatty acid biosynthesis genes (Krogan *et al.* 2004; Lindstrom *et al.* 2006; Cheng *et al.* 2015). Indeed, our work identified an increase in mRNA expression of *FAS1* and *FAS2* in *sec14-1^ts^eaf1*Δ cells, which may reflect the cells’ attempts at compensating for decreased lipogenesis flux upon deletion of *EAF1* (Figure 3). NuA4 has also been implicated in the inhibition of AMPK1/Snf1, and NuA4 mutants display hyperactivated Snf1 (Lu *et al.* 2011). As Snf1 phosphorylates and inhibits the activity of acetyl-CoA carboxylase (Acc1), the first step of fatty acid *de novo* synthesis (Hofbauer *et al.* 2014), NuA4 maybe regulating fatty acid biosynthesis pathway by reducing Snf1 activity and maintaining Acc1 activity. Indeed, activity of Acc1 is reduced in WCEs from NuA4 mutants (Rollins *et al.* 2017). Alternatively, and perhaps not mutually exclusively, NuA4 may also have potential targets within the fatty acid biosynthesis pathway. In fact, lysine acetylation of proteins in this pathway, including Fas1 and Fas2, have been detected in both yeast and higher organisms (Choudhary *et al.* 2009; Henriksen *et al.* 2012; Weinert *et al.* 2014; Downey *et al.* 2015; Madsen *et al.* 2015). While the mechanism(s) through which NuA4 is contributing to cellular lipid homeostasis remain unclear, our work shows that NuA4 has underappreciated roles in lipid homeostasis.

## Supplementary Material

Supplemental material is available online at www.g3journal.org/lookup/suppl/doi:10.1534/g3.117.041053/-/DC1.

## References

[bib1] Alfaro G. Johansen J. Dighe S. A. Duamel G. Kozminski K. G., 2011 The sterol-binding protein Kes1/Osh4p is a regulator of polarized exocytosis. Traffic 12: 1521–1536. 21819498 10.1111/j.1600-0854.2011.01265.x

[bib2] Ambroziak J. Henry S. A., 1994 INO2 and INO4 gene products, positive regulators of phospholipid biosynthesis in Saccharomyces cerevisiae, form a complex that binds to the INO1 promoter. J. Biol. Chem. 269: 15344–15349. 8195172

[bib3] Ashburner B. P. Lopes J. M., 1995 Autoregulated expression of the yeast INO2 and INO4 helix-loop-helix activator genes effects cooperative regulation on their target genes. Mol. Cell. Biol. 15: 1709–1715. 7862162 10.1128/mcb.15.3.1709 PMC230395

[bib4] Audhya A. Emr S. D., 2003 Regulation of PI4,5P2 synthesis by nuclear-cytoplasmic shuttling of the Mss4 lipid kinase. EMBO J. 22: 4223–4236. 12912920 10.1093/emboj/cdg397 PMC175787

[bib5] Auger A. Galarneau L. Altaf M. Nourani A. Doyon Y., 2008 Eaf1 is the platform for NuA4 molecular assembly that evolutionarily links chromatin acetylation to ATP-dependent exchange of histone H2A variants. Mol. Cell. Biol. 28: 2257–2270. 18212047 10.1128/MCB.01755-07 PMC2268442

[bib6] Brachmann C. B. Davies A. Cost G. J. Caputo E. Li J., 1998 Designer deletion strains derived from *Saccharomyces cerevisiae* S288C: a useful set of strains and plasmids for PCR-mediated gene disruption and other applications. Yeast 14: 115–132. 9483801 10.1002/(SICI)1097-0061(19980130)14:2<115::AID-YEA204>3.0.CO;2-2

[bib7] Chang H. J. Jones E. W. Henry S. A., 2002 Role of the unfolded protein response pathway in regulation of *INO1* and in the *sec14* bypass mechanism in *Saccharomyces cerevisiae*. Genetics 162: 29–43. 12242221 10.1093/genetics/162.1.29 PMC1462253

[bib8] Cheng X. Auger A. Altaf M. Drouin S. Paquet E., 2015 Eaf1 links the NuA4 histone acetyltransferase complex to Htz1 incorporation and regulation of purine biosynthesis. Eukaryot. Cell 14: 535–544. 25841019 10.1128/EC.00004-15 PMC4452573

[bib9] Cherry J. M. Hong E. L. Amundsen C. Balakrishnan R. Binkley G., 2012 *Saccharomyces* Genome Database: the genomics resource of budding yeast. Nucleic Acids Res. 40: D700–D705. 22110037 10.1093/nar/gkr1029 PMC3245034

[bib10] Choudhary C. Kumar C. Gnad F. Nielsen M. L. Rehman M., 2009 Lysine acetylation targets protein complexes and co-regulates major cellular functions. Science 325: 834–840. 19608861 10.1126/science.1175371

[bib11] Clarke A. S. Lowell J. E. Jacobson S. J. Pillus L., 1999 Esa1p is an essential histone acetyltransferase required for cell cycle progression. Mol. Cell. Biol. 19: 2515–2526. 10082517 10.1128/mcb.19.4.2515 PMC84044

[bib12] Culbertson M. R. Henry S. A., 1975 Inositol-requiring mutants of *Saccharomyces cerevisiae*. Genetics 80: 23–40. 1093935 10.1093/genetics/80.1.23 PMC1213318

[bib13] Curwin A. J. Fairn G. D. McMaster C. R., 2009 Phospholipid transfer protein Sec14 is required for trafficking from endosomes and regulates distinct trans-Golgi export pathways. J. Biol. Chem. 284: 7364–7375. 19129178 10.1074/jbc.M808732200 PMC2652273

[bib14] Downey M. Baetz K., 2016 Building a KATalogue of acetyllysine targeting and function. Brief. Funct. Genomics 15: 109–118. 26512033 10.1093/bfgp/elv045 PMC4803063

[bib15] Downey M. Johnson J. R. Davey N. E. Newton B. W. Johnson T. L., 2015 Acetylome profiling reveals overlap in the regulation of diverse processes by sirtuins, gcn5, and esa1. Mol. Cell. Proteomics 14: 162–176. 25381059 10.1074/mcp.M114.043141 PMC4288252

[bib16] Doyon Y. Cote J., 2004 The highly conserved and multifunctional NuA4 HAT complex. Curr. Opin. Genet. Dev. 14: 147–154. 15196461 10.1016/j.gde.2004.02.009

[bib17] Drazic A. Myklebust L. M. Ree R. Arnesen T., 2016 The world of protein acetylation. Biochim. Biophys. Acta 1864: 1372–1401. 27296530 10.1016/j.bbapap.2016.06.007

[bib18] Eisenberg T. Buttner S., 2014 Lipids and cell death in yeast. FEMS Yeast Res. 14: 179–197. 24119111 10.1111/1567-1364.12105 PMC4255311

[bib19] Engel S. R. Dietrich F. S. Fisk D. G. Binkley G. Balakrishnan R., 2014 The reference genome sequence of *Saccharomyces cerevisiae*: then and now. G3 (Bethesda) 4: 389–398. 24374639 10.1534/g3.113.008995 PMC3962479

[bib20] Fairn G. D. Curwin A. J. Stefan C. J. McMaster C. R., 2007 The oxysterol binding protein Kes1p regulates Golgi apparatus phosphatidylinositol-4-phosphate function. Proc. Natl. Acad. Sci. USA 104: 15352–15357. 17881569 10.1073/pnas.0705571104 PMC2000554

[bib21] Fang M. Kearns B. G. Gedvilaite A. Kagiwada S. Kearns M., 1996 Kes1p shares homology with human oxysterol binding protein and participates in a novel regulatory pathway for yeast Golgi-derived transport vesicle biogenesis. EMBO J. 15: 6447–6459. 8978672 PMC452470

[bib22] Feddersen S. Neergaard T. B. Knudsen J. Faergeman N. J., 2007 Transcriptional regulation of phospholipid biosynthesis is linked to fatty acid metabolism by an acyl-CoA-binding-protein-dependent mechanism in *Saccharomyces cerevisiae*. Biochem. J. 407: 219–230. 17593018 10.1042/BJ20070315 PMC2049021

[bib23] Fei W. Shui G. Zhang Y. Krahmer N. Ferguson C., 2011 A role for phosphatidic acid in the formation of “supersized” lipid droplets. PLoS Genet. 7: e1002201. 21829381 10.1371/journal.pgen.1002201 PMC3145623

[bib24] Fernandez-Murray J. P. McMaster C. R., 2016 Lipid synthesis and membrane contact sites: a crossroads for cellular physiology. J. Lipid Res. 57: 1789–1805. 27521373 10.1194/jlr.R070920 PMC5036376

[bib25] Fyrst H. Oskouian B. Kuypers F. A. Saba J. D., 1999 The *PLB2* gene of *Saccharomyces cerevisiae* confers resistance to lysophosphatidylcholine and encodes a phospholipase B/lysophospholipase. Biochemistry 38: 5864–5871. 10231538 10.1021/bi9824590

[bib26] Gaspar M. L. Hofbauer H. F. Kohlwein S. D. Henry S. A., 2011 Coordination of storage lipid synthesis and membrane biogenesis: evidence for cross-talk between triacylglycerol metabolism and phosphatidylinositol synthesis. J. Biol. Chem. 286: 1696–1708. 20972264 10.1074/jbc.M110.172296 PMC3023464

[bib27] Ghosh R. Bankaitis V. A., 2011 Phosphatidylinositol transfer proteins: negotiating the regulatory interface between lipid metabolism and lipid signaling in diverse cellular processes. Biofactors 37: 290–308. 21915936 10.1002/biof.180

[bib28] Greenberg M. L. Reiner B. Henry S. A., 1982 Regulatory mutations of inositol biosynthesis in yeast: isolation of inositol-excreting mutants. Genetics 100: 19–33. 7047296 10.1093/genetics/100.1.19 PMC1201799

[bib29] Hama H. Schnieders E. A. Thorner J. Takemoto J. Y. DeWald D. B., 1999 Direct involvement of phosphatidylinositol 4-phosphate in secretion in the yeast *Saccharomyces cerevisiae*. J. Biol. Chem. 274: 34294–34300. 10567405 10.1074/jbc.274.48.34294

[bib30] Hancock L. C. Behta R. P. Lopes J. M., 2006 Genomic analysis of the Opi- phenotype. Genetics 173: 621–634. 16582425 10.1534/genetics.106.057489 PMC1526532

[bib31] Henriksen P. Wagner S. A. Weinert B. T. Sharma S. Bacinskaja G., 2012 Proteome-wide analysis of lysine acetylation suggests its broad regulatory scope in *Saccharomyces cerevisiae*. Mol. Cell. Proteomics 11: 1510–1522. 22865919 10.1074/mcp.M112.017251 PMC3494197

[bib32] Henry S. A. Kohlwein S. D. Carman G. M., 2012 Metabolism and regulation of glycerolipids in the yeast *Saccharomyces cerevisiae*. Genetics 190: 317–349. 22345606 10.1534/genetics.111.130286 PMC3276621

[bib33] Hirsch J. P. Henry S. A., 1986 Expression of the Saccharomyces cerevisiae inositol-1-phosphate synthase (INO1) gene is regulated by factors that affect phospholipid synthesis. Mol. Cell. Biol. 6: 3320–3328. 3025587 10.1128/mcb.6.10.3320-3328.1986 PMC367077

[bib34] Hofbauer H. F. Schopf F. H. Schleifer H. Knittelfelder O. L. Pieber B., 2014 Regulation of gene expression through a transcriptional repressor that senses acyl-chain length in membrane phospholipids. Dev. Cell 29: 729–739. 24960695 10.1016/j.devcel.2014.04.025 PMC4070385

[bib35] Huang D. W. Sherman B. T. Lempicki R. A., 2009 Bioinformatics enrichment tools: paths toward the comprehensive functional analysis of large gene lists. Nucleic Acids Res. 37: 1–13. 19033363 10.1093/nar/gkn923 PMC2615629

[bib36] Jackson C. L. Bouvet S., 2014 Arfs at a glance. J. Cell Sci. 127: 4103–4109. 25146395 10.1242/jcs.144899

[bib37] Kawaguchi A. Tomoda H. Okuda S. Awaya J. Omura S., 1979 Cerulenin resistance in a cerulenin-producing fungus. Isolation of cerulenin insensitive fatty acid synthetase. Arch. Biochem. Biophys. 197: 30–35. 575612 10.1016/0003-9861(79)90214-5

[bib38] Kawaguchi A. Tomoda H. Nozoe S. Omura S. Okuda S., 1982 Mechanism of action of cerulenin on fatty acid synthetase. Effect of cerulenin on iodoacetamide-induced malonyl-CoA decarboxylase activity. J. Biochem. 92: 7–12. 6749834 10.1093/oxfordjournals.jbchem.a133933

[bib39] Koffel R. Tiwari R. Falquet L. Schneiter R., 2005 The *Saccharomyces cerevisiae YLL012/YEH1*, *YLR020/YEH2*, and *TGL1* genes encode a novel family of membrane-anchored lipases that are required for steryl ester hydrolysis. Mol. Cell. Biol. 25: 1655–1668. 15713625 10.1128/MCB.25.5.1655-1668.2005 PMC549362

[bib40] Konarzewska P. Esposito M. Shen C. H., 2012 INO1 induction requires chromatin remodelers Ino80p and Snf2p but not the histone acetylases. Biochem. Biophys. Res. Commun. 418: 483–488. 22281492 10.1016/j.bbrc.2012.01.044

[bib41] Krogan N. J. Baetz K. Keogh M. C. Datta N. Sawa C., 2004 Regulation of chromosome stability by the histone H2A variant Htz1, the Swr1 chromatin remodeling complex, and the histone acetyltransferase NuA4. Proc. Natl. Acad. Sci. USA 101: 13513–13518. 15353583 10.1073/pnas.0405753101 PMC518788

[bib42] Kuziora M. A. Chalmers J. H. Jr Douglas M. G. Hitzeman R. A. Mattick J. S., 1983 Molecular cloning of fatty acid synthetase genes from Saccharomyces cerevisiae. J. Biol. Chem. 258: 11648–11653. 6311818

[bib43] LeBlanc M. A. McMaster C. R., 2010 Lipid binding requirements for oxysterol-binding protein Kes1 inhibition of autophagy and endosome-trans-Golgi trafficking pathways. J. Biol. Chem. 285: 33875–33884. 20729555 10.1074/jbc.M110.147264 PMC2962487

[bib44] Li X. Rivas M. P. Fang M. Marchena J. Mehrotra B., 2002 Analysis of oxysterol binding protein homologue Kes1p function in regulation of Sec14p-dependent protein transport from the yeast Golgi complex. J. Cell Biol. 157: 63–77. 11916983 10.1083/jcb.200201037 PMC2173257

[bib45] Lindstrom K. C. Vary J. C. Jr Parthun M. R. Delrow J. Tsukiyama T., 2006 Isw1 functions in parallel with the NuA4 and Swr1 complexes in stress-induced gene repression. Mol. Cell. Biol. 26: 6117–6129. 16880522 10.1128/MCB.00642-06 PMC1592817

[bib46] Loewen C. J. Roy A. Levine T. P., 2003 A conserved ER targeting motif in three families of lipid binding proteins and in Opi1p binds VAP. EMBO J. 22: 2025–2035. 12727870 10.1093/emboj/cdg201 PMC156073

[bib47] Loewen C. J. Gaspar M. L. Jesch S. A. Delon C. Ktistakis N. T., 2004 Phospholipid metabolism regulated by a transcription factor sensing phosphatidic acid. Science 304: 1644–1647. 15192221 10.1126/science.1096083

[bib48] Longtine M. S. McKenzie A. III Demarini D. J. Shah N. G. Wach A., 1998 Additional modules for versatile and economical PCR-based gene deletion and modification in *Saccharomyces cerevisiae*. Yeast 14: 953–961. 9717241 10.1002/(SICI)1097-0061(199807)14:10<953::AID-YEA293>3.0.CO;2-U

[bib49] Love M. I. Huber W. Anders S., 2014 Moderated estimation of fold change and dispersion for RNA-seq data with DESeq2. Genome Biol. 15: 550. 25516281 10.1186/s13059-014-0550-8 PMC4302049

[bib50] Lu J. Y. Lin Y. Y. Sheu J. C. Wu J. T. Lee F. J., 2011 Acetylation of yeast AMPK controls intrinsic aging independently of caloric restriction. Cell 146: 969–979. 21906795 10.1016/j.cell.2011.07.044 PMC3176974

[bib51] Lu P. Y. Levesque N. Kobor M. S., 2009 NuA4 and SWR1-C: two chromatin-modifying complexes with overlapping functions and components. Biochem. Cell Biol. 87: 799–815. 19898529 10.1139/O09-062

[bib52] Madsen C. T. Sylvestersen K. B. Young C. Larsen S. C. Poulsen J. W., 2015 Biotin starvation causes mitochondrial protein hyperacetylation and partial rescue by the SIRT3-like deacetylase Hst4p. Nat. Commun. 6: 7726. 26158509 10.1038/ncomms8726 PMC4510963

[bib53] McGee T. P. Skinner H. B. Whitters E. A. Henry S. A. Bankaitis V. A., 1994 A phosphatidylinositol transfer protein controls the phosphatidylcholine content of yeast Golgi membranes. J. Cell Biol. 124: 273–287. 8294512 10.1083/jcb.124.3.273 PMC2119930

[bib54] Menzies K. J. Zhang H. Katsyuba E. Auwerx J., 2016 Protein acetylation in metabolism — metabolites and cofactors. Nat. Rev. Endocrinol. 12: 43–60. 26503676 10.1038/nrendo.2015.181

[bib55] Merkel O. Fido M. Mayr J. A. Pruger H. Raab F., 1999 Characterization and function *in vivo* of two novel phospholipases B/lysophospholipases from *Saccharomyces cerevisiae*. J. Biol. Chem. 274: 28121–28127. 10497163 10.1074/jbc.274.40.28121

[bib56] Mitchell L. Lambert J. P. Gerdes M. Al-Madhoun A. S. Skerjanc I. S., 2008 Functional dissection of the NuA4 histone acetyltransferase reveals its role as a genetic hub and that Eaf1 is essential for complex integrity. Mol. Cell. Biol. 28: 2244–2256. 18212056 10.1128/MCB.01653-07 PMC2268438

[bib57] Mitchell L. Lau A. Lambert J. P. Zhou H. Fong Y., 2011 Regulation of septin dynamics by the *Saccharomyces cerevisiae* lysine acetyltransferase NuA4. PLoS One 6: e25336. 21984913 10.1371/journal.pone.0025336 PMC3184947

[bib58] Mora G. Scharnewski M. Fulda M., 2012 Neutral lipid metabolism influences phospholipid synthesis and deacylation in *Saccharomyces cerevisiae*. PLoS One 7: e49269. 23139841 10.1371/journal.pone.0049269 PMC3489728

[bib59] Mullner H. Daum G., 2004 Dynamics of neutral lipid storage in yeast. Acta Biochim. Pol. 51: 323–347. 15218532

[bib60] Nourani A. Utley R. T. Allard S. Cote J., 2004 Recruitment of the NuA4 complex poises the PHO5 promoter for chromatin remodeling and activation. EMBO J. 23: 2597–2607. 15175650 10.1038/sj.emboj.7600230 PMC449761

[bib61] Oelkers P. Cromley D. Padamsee M. Billheimer J. T. Sturley S. L., 2002 The DGA1 gene determines a second triglyceride synthetic pathway in yeast. J. Biol. Chem. 277: 8877–8881. 11751875 10.1074/jbc.M111646200

[bib62] Olkkonen V. M., 2013 OSBP-related proteins: liganding by glycerophospholipids opens new insight into their function. Molecules 18: 13666–13679. 24196413 10.3390/molecules181113666 PMC6270239

[bib63] Patton-Vogt J. L. Griac P. Sreenivas A. Bruno V. Dowd S., 1997 Role of the yeast phosphatidylinositol/phosphatidylcholine transfer protein (Sec14p) in phosphatidylcholine turnover and INO1 regulation. J. Biol. Chem. 272: 20873–20883. 9252414 10.1074/jbc.272.33.20873

[bib64] Pfaffl M. W., 2001 A new mathematical model for relative quantification in real-time RT-PCR. Nucleic Acids Res. 29: e45. 11328886 10.1093/nar/29.9.e45 PMC55695

[bib65] Quinlan A. R. Hall I. M., 2010 BEDTools: a flexible suite of utilities for comparing genomic features. Bioinformatics 26: 841–842. 20110278 10.1093/bioinformatics/btq033 PMC2832824

[bib66] Reid J. L. Iyer V. R. Brown P. O. Struhl K., 2000 Coordinate regulation of yeast ribosomal protein genes is associated with targeted recruitment of Esa1 histone acetylase. Mol. Cell 6: 1297–1307. 11163204 10.1016/s1097-2765(00)00128-3

[bib67] Ricicova M. Hamidi M. Quiring A. Niemisto A. Emberly E., 2013 Dissecting genealogy and cell cycle as sources of cell-to-cell variability in MAPK signaling using high-throughput lineage tracking. Proc. Natl. Acad. Sci. USA 110: 11403–11408. 23803859 10.1073/pnas.1215850110 PMC3710830

[bib68] Rollins M. Huard S. Morettin A. Takuski J. Pham T. T., 2017 Lysine acetyltransferase NuA4 and acetyl-CoA regulate glucose-deprived stress granule formation in *Saccharomyces cerevisiae*. PLoS Genet. 13: e1006626. 28231279 10.1371/journal.pgen.1006626 PMC5344529

[bib69] Roney I. J. Rudner A. D. Couture J. F. Kaern M., 2016 Improvement of the reverse tetracycline transactivator by single amino acid substitutions that reduce leaky target gene expression to undetectable levels. Sci. Rep. 6: 27697. 27323850 10.1038/srep27697 PMC4914848

[bib70] Salas-Santiago B. Lopes J. M., 2014 *Saccharomyces cerevisiae* essential genes with an Opi^−^ phenotype. G3 (Bethesda) 4: 761–767. 24558266 10.1534/g3.113.010140 PMC4059245

[bib71] Sandager L. Gustavsson M. H. Stahl U. Dahlqvist A. Wiberg E., 2002 Storage lipid synthesis is non-essential in yeast. J. Biol. Chem. 277: 6478–6482. 11741946 10.1074/jbc.M109109200

[bib72] Santiago T. C. Mamoun C. B., 2003 Genome expression analysis in yeast reveals novel transcriptional regulation by inositol and choline and new regulatory functions for Opi1p, Ino2p, and Ino4p. J. Biol. Chem. 278: 38723–38730. 12871953 10.1074/jbc.M303008200

[bib73] Schaaf G. Ortlund E. A. Tyeryar K. R. Mousley C. J. Ile K. E., 2008 Functional anatomy of phospholipid binding and regulation of phosphoinositide homeostasis by proteins of the sec14 superfamily. Mol. Cell 29: 191–206. 18243114 10.1016/j.molcel.2007.11.026 PMC7808562

[bib74] Shen H. Dowhan W., 1996 Reduction of CDP-diacylglycerol synthase activity results in the excretion of inositol by *Saccharomyces cerevisiae*. J. Biol. Chem. 271: 29043–29048. 8910557 10.1074/jbc.271.46.29043

[bib75] Shen H. Dowhan W., 1997 Regulation of phospholipid biosynthetic enzymes by the level of CDP-diacylglycerol synthase activity. J. Biol. Chem. 272: 11215–11220. 9111022 10.1074/jbc.272.17.11215

[bib76] Shen H. Heacock P. N. Clancey C. J. Dowhan W., 1996 The *CDS1* gene encoding CDP-diacylglycerol synthase in *Saccharomyces cerevisiae* is essential for cell growth. J. Biol. Chem. 271: 789–795. 8557688 10.1074/jbc.271.2.789

[bib77] Shirra M. K. Patton-Vogt J. Ulrich A. Liuta-Tehlivets O. Kohlwein S. D., 2001 Inhibition of acetyl coenzyme A carboxylase activity restores expression of the *INO1* gene in a *snf1* mutant strain of *Saccharomyces cerevisiae*. Mol. Cell. Biol. 21: 5710–5722. 11486011 10.1128/MCB.21.17.5710-5722.2001 PMC87291

[bib78] Suka N. Suka Y. Carmen A. A. Wu J. Grunstein M., 2001 Highly specific antibodies determine histone acetylation site usage in yeast heterochromatin and euchromatin. Mol. Cell 8: 473–479. 11545749 10.1016/s1097-2765(01)00301-x

[bib79] Tripathi A. Nile A. H. Bankaitis V. A., 2014 Sec14-like phosphatidylinositol-transfer proteins and diversification of phosphoinositide signalling outcomes. Biochem. Soc. Trans. 42: 1383–1388. 25233419 10.1042/BST20140187 PMC5470545

[bib80] Villa-Garcia M. J. Choi M. S. Hinz F. I. Gaspar M. L. Jesch S. A., 2011 Genome-wide screen for inositol auxotrophy in *Saccharomyces cerevisiae* implicates lipid metabolism in stress response signaling. Mol. Genet. Genomics 285: 125–149. 21136082 10.1007/s00438-010-0592-x PMC3037835

[bib81] Wang C. W., 2015 Lipid droplet dynamics in budding yeast. Cell. Mol. Life Sci. 72: 2677–2695. 25894691 10.1007/s00018-015-1903-5 PMC11113813

[bib82] Weinert B. T. Iesmantavicius V. Moustafa T. Scholz C. Wagner S. A., 2014 Acetylation dynamics and stoichiometry in *Saccharomyces cerevisiae*. Mol. Syst. Biol. 10: 716. 24489116 10.1002/msb.134766 PMC4023402

[bib83] White M. J. Hirsch J. P. Henry S. A., 1991 The OPI1 gene of Saccharomyces cerevisiae, a negative regulator of phospholipid biosynthesis, encodes a protein containing polyglutamine tracts and a leucine zipper. J. Biol. Chem. 266: 863–872. 1985968

[bib84] Xie Z. Fang M. Bankaitis V. A., 2001 Evidence for an intrinsic toxicity of phosphatidylcholine to Sec14p-dependent protein transport from the yeast Golgi complex. Mol. Biol. Cell 12: 1117–1129. 11294911 10.1091/mbc.12.4.1117 PMC32291

