# Supplementary material for: NuA4 Lysine Acetyltransferase Complex Contributes to Phospholipid Homeostasis in Saccharomyces cerevisiae
Source: G3 (Bethesda). 2017 Apr 28;7(6):1799–809. doi: 10.1534/g3.117.041053 (PMC5473759; doi:10.1534/g3.117.041053)
Supplement: Supplementary file 2 [file 1799FileS2.pdf]

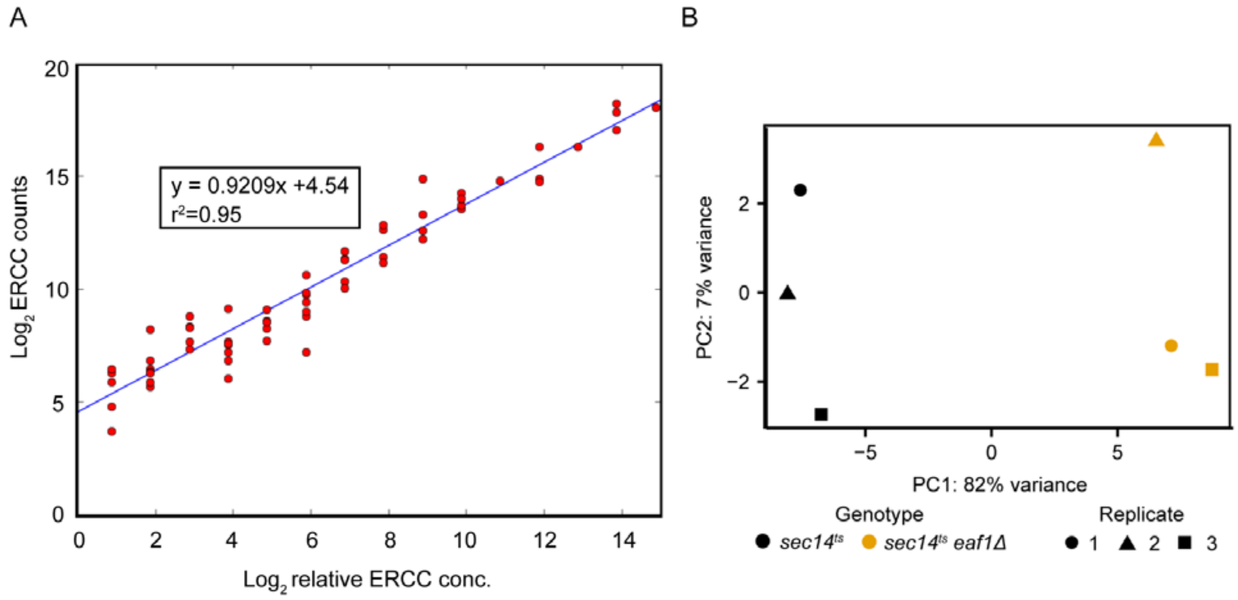

Figure S1. ERCC standard validation and principal component analysis for RNA-seq samples. A) Representative ERCC standard curve. Graph shows standard curve calculated for one biological replicate of *sec14-1*^{ts}. Log_2 transformed ERCC counts are plotted on the y-axis while Log_2 transformed ERCC concentrations are plotted on the x-axis. **B)** Principal component analysis of the normalized gene counts. The graph plots the first two principal components. As expected, the RNA-seq datasets separate by genotype on PC1, demonstrating that the majority of variance is explained by genotype and not by biological replicate.

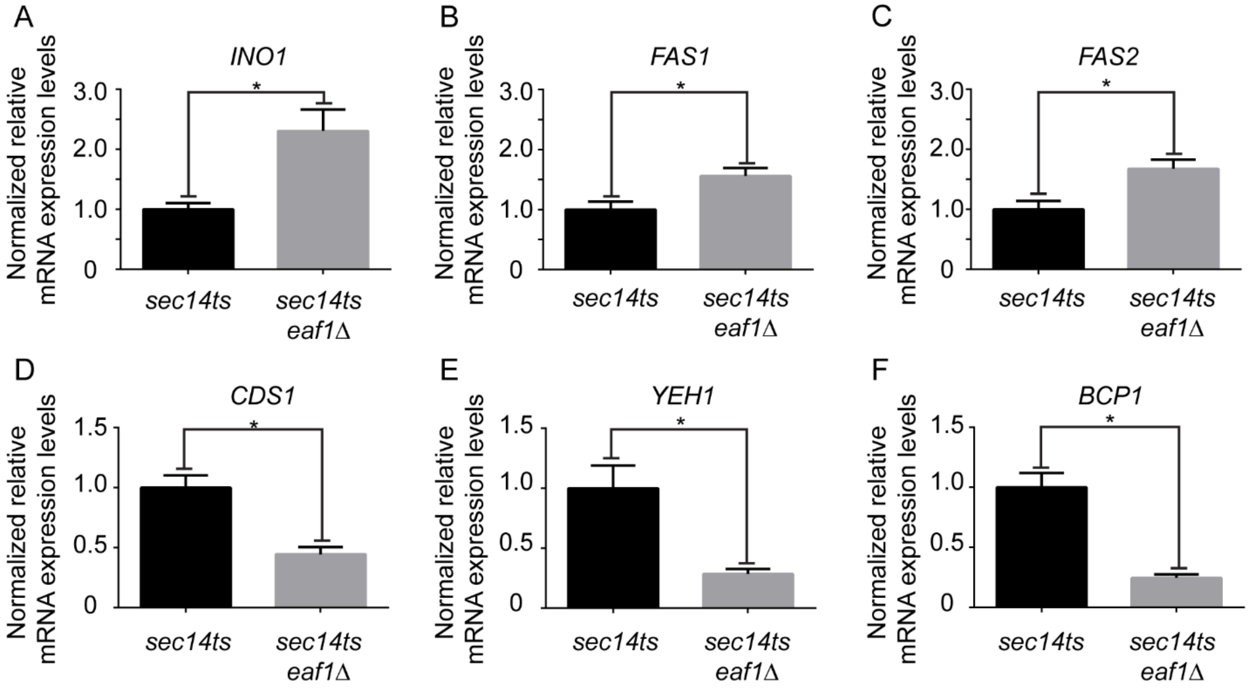

Figure S2. Confirmation of mRNA expression of lipid metabolic genes identified in transcriptome comparison of *sec14-1^{ts}eaf1Δ* and *sec14-1^{ts}*. Strains were grown to log phase in YPD at 30°C then shifted to 33.5°C for two hours prior to RNA extraction. mRNA expression levels were normalized to *TDH3* and *ACT1* expression. The mean normalized Log_2 fold expression (+S.E.M.) from three biological replicates is shown. Statistical analysis was performed by unpaired two-tailed t-test: *p-value ≤0.05.

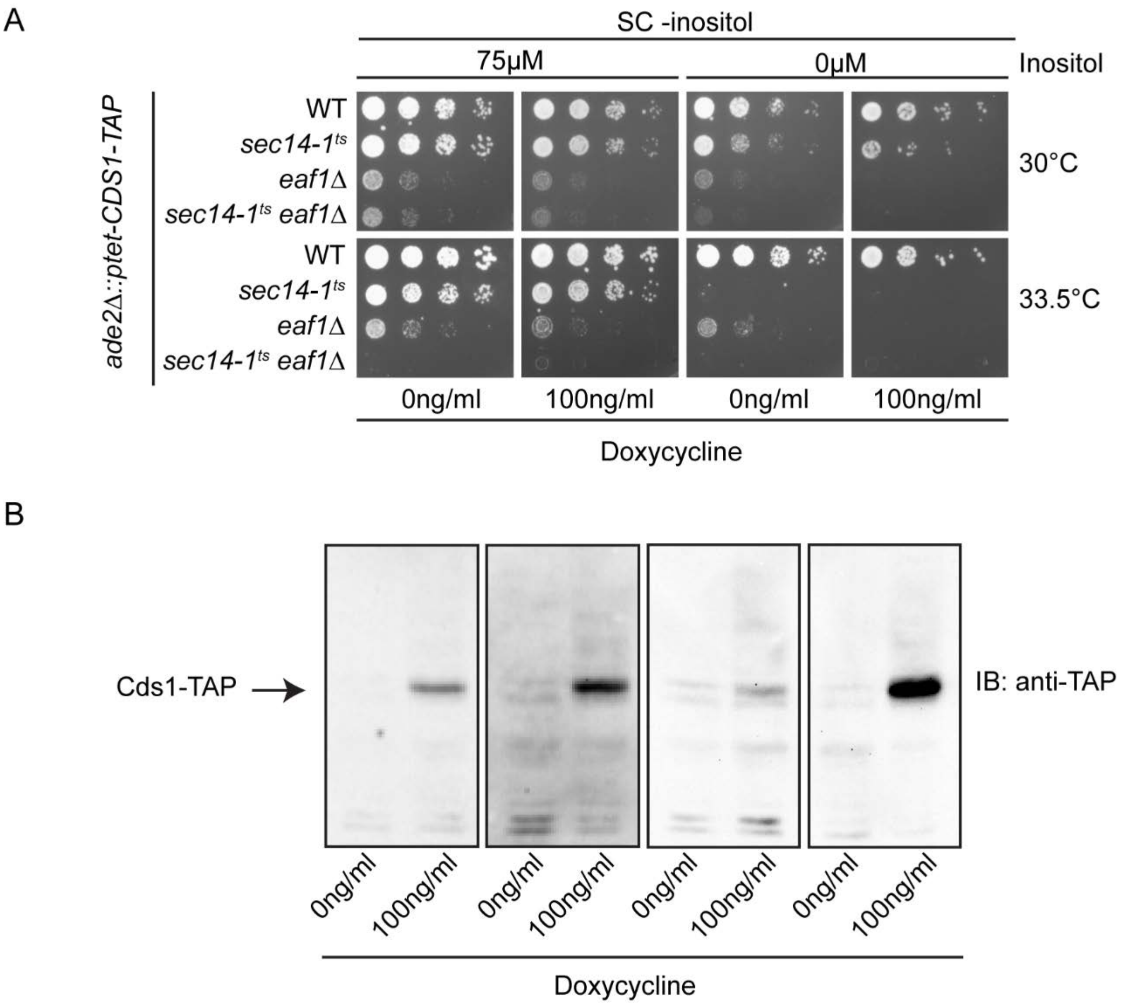

Figure S3. *CDS1* overexpression does not rescue growth defects of *sec14-1^{ts}eaf1Δ*. **A)** Overexpression of *CDS1* does not improve growth of *sec14-1^{ts}eaf1Δ* in inositol depleted conditions. Culture of strains containing Integrated TAP-tagged *CDS1* gene under a doxycycline-inducible promoter in wildtype (YKB4325), *sec14-1^{ts}*(YKB4326), *eaf1Δ* (YKB4327), and *sec14-1^{ts}eaf1Δ* (YKB4328) backgrounds were grown to mid-log phase prior to being diluted to an OD_{600} of 0.1 and 4 times 10-fold serial dilutions were spotted on SC-inositol media supplemented with the indicated amount of myo-inositol and doxycycline. Plates were incubated for two days at 30°C or 33.5°C and images are representative of three biological replicates. **B)** *CDS1* doxycycline-inducible protein expression confirmation by immunoblot. Whole cell extracts from strains listed in A) were collected from cultures grown to mid-log phase at 30°C with or without doxycycline. 12 μg of WCE was subsequently resolved on 7.5% SDS-PAGE gel, and subjected to immunoblot analysis with α-TAP antibodies.

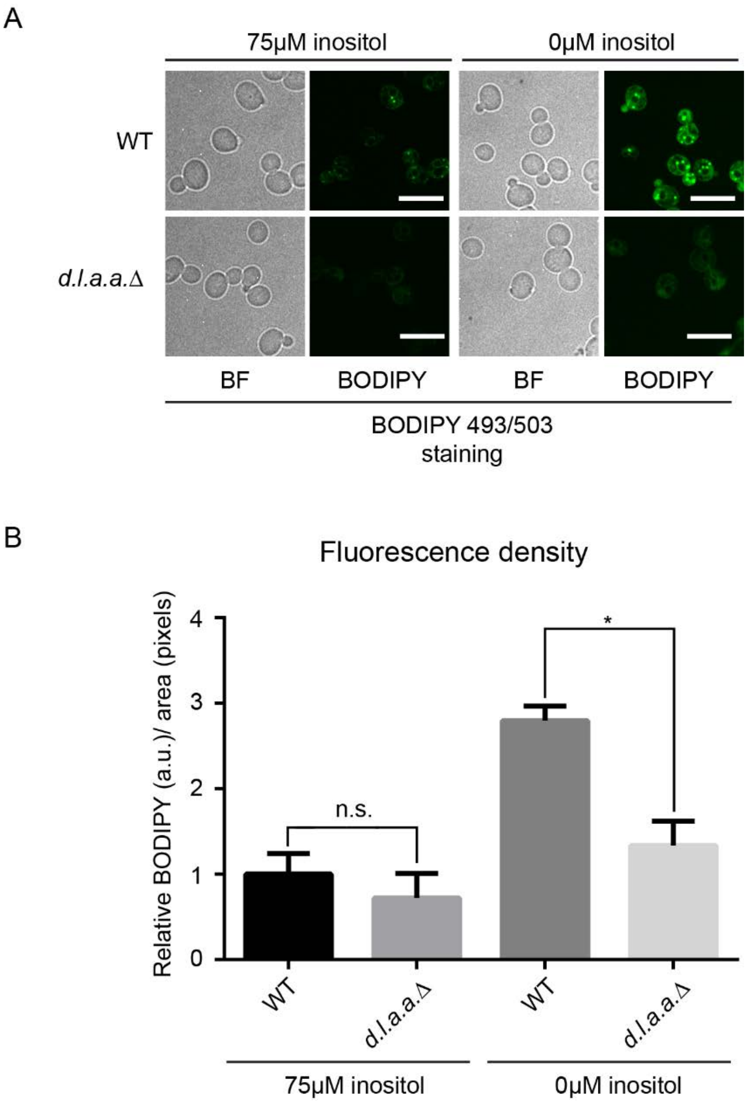

Figure S4. Lipid droplet staining is significantly impaired in strains lacking neutral lipid biosynthetic genes. A) Wildtype (YKB1079), and *dga1Δlro1Δare1Δare2Δ* (*d.l.a.a.Δ;* YKB4336) were grown to mid-log phase in SC media at 30°C before shifting to inositol-supplemented (75 µM myo-inositol) SC media or inositol-depleted SC media for 2 hours at 30°C prior to being stained with 10 µM BOPIDY® 493/503 for 10 min before imaging. Images shown are mid-field view of representative cells for each sample (Scale bar =10 µm). **B)** Graphic display of the mean relative fluorescence density for each strain and condition. Cells were first segmented from brightfield images using a custom Matlab script then fluorescence was measured for each cell using the outlines (see Materials and Methods for details). Mean fluorescence density was measured from 100 cells across three biological replicates relative to WT fluorescence in inositol supplemented media (+S.E.M.). Statistical analysis was performed by one-way ANOVA with Tukey's multiple comparisons test: *p-value <0.05; n.s. not significant.

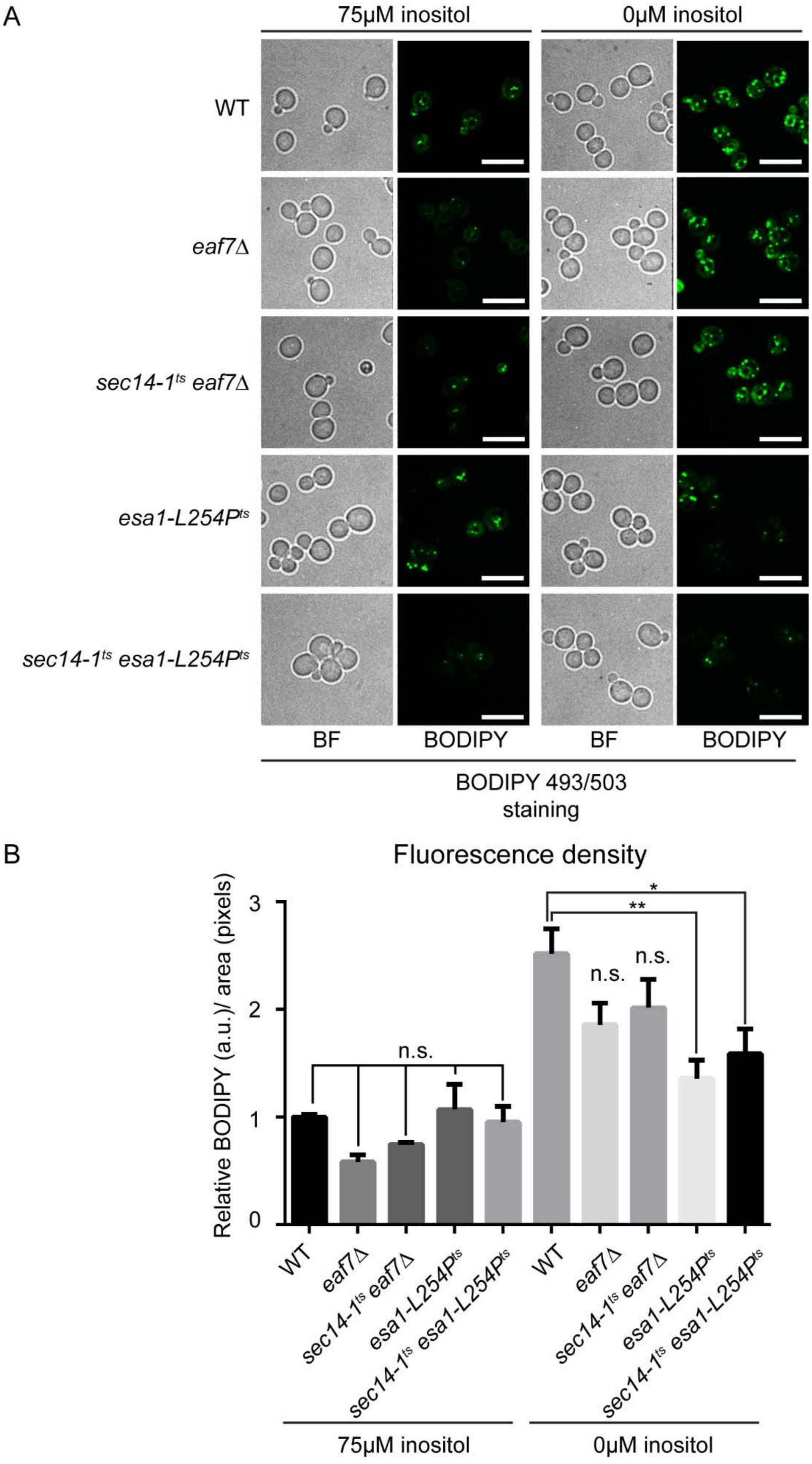

A
75μM inositol
0μM inositol
WT
eaf7Δ
sec14-1ts eaf7Δ
esa1-L254Pts
sec14-1ts esa1-L254Pts
BF
BODIPY
BF
BODIPY
BODIPY 493/503 staining
B
Fluorescence density
Relative BODIPY (a.u.)/ area (pixels)
3
2
1
0
n.s.
n.s.
n.s.
**
*
WT
eaf7Δ
sec14-1ts eaf7Δ
esa1-L254Pts
sec14-1ts esa1-L254Pts
WT
eaf7Δ
sec14-1ts eaf7Δ
esa1-L254Pts
sec14-1ts esa1-L254Pts
75μM inositol
0μM inositol

Figure S5. Lipid droplet dynamics is impaired in *EAF7* and *ESA1* mutants. Wildtype (YKB1079), *eaf7*Δ (YKB3292), *sec14-1*^{ts}*eaf7*Δ (YKB4068), *esa1-L254P*^{ts} (YKB4236), and *sec14-1*^{ts}*esa1-L254P*^{ts} (YKB4242) were grown to mid-log phase in SC media at 30°C before shifting to inositol-supplemented (75 µM myo-inositol) SC media or inositol-depleted SC media for 2 hours at 30°C prior to being stained with 10 µM BOPIDY® 493/503 for 10 min before imaging. Images shown are mid-field view of representative cells for each sample (Scale bar =10 µm). **B)** Graphic display of the mean relative fluorescence density for each strain and condition. Cells were first segmented from brightfield images using a custom Matlab script then fluorescence was measured for each cell using the outlines (see Materials and Methods for details). Mean fluorescence density was measured from 100 cells across three biological replicates relative to WT fluorescence in inositol supplemented media (+S.E.M.). Statistical analysis was performed by one-way ANOVA with Tukey's multiple comparisons test: *p-value <0.05; n.s. not significant.

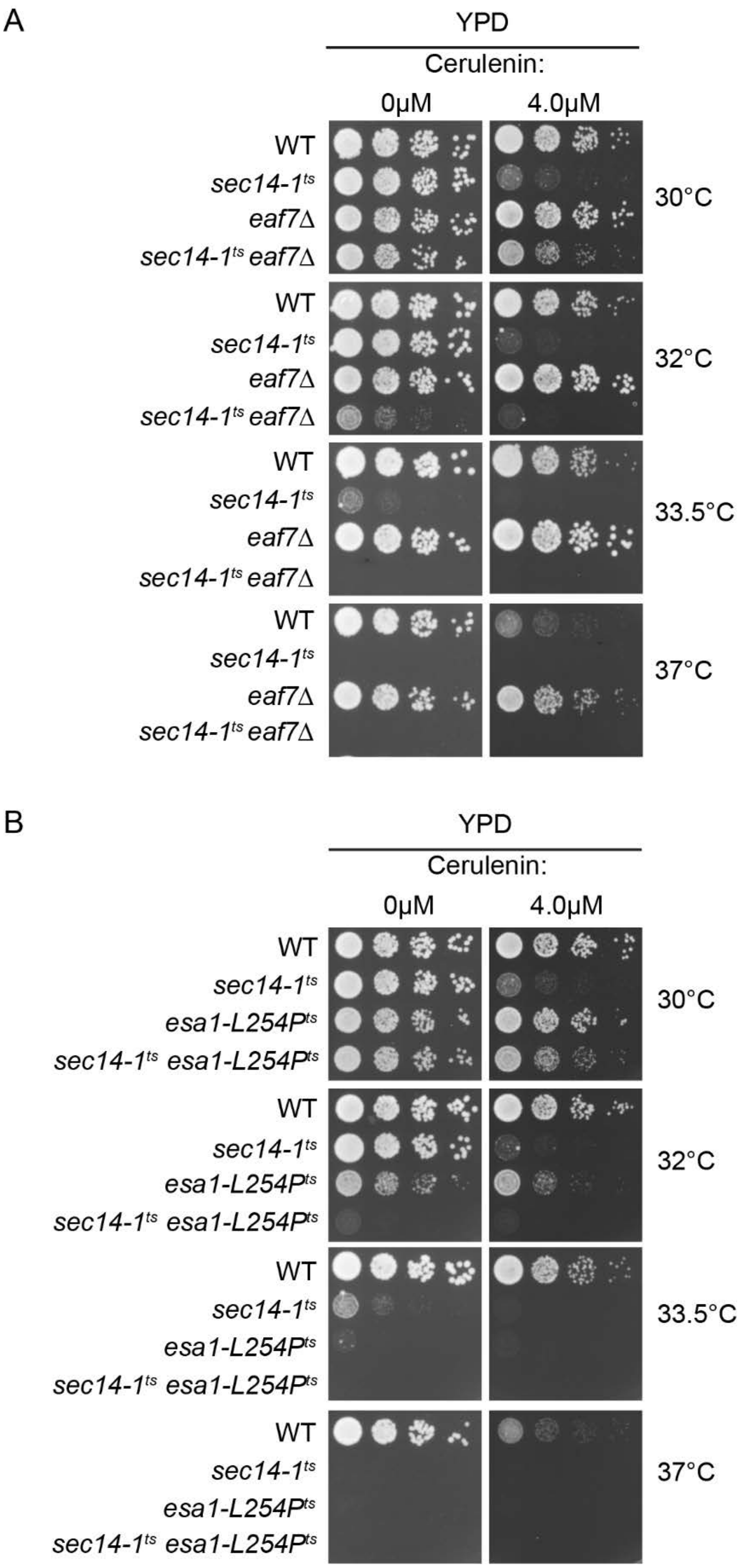
A
YPD
Cerulenin:
0μM
4.0μM
WT
sec14-1ts
eaf7Δ
sec14-1ts eaf7Δ
30°C
32°C
33.5°C
37°C
B
YPD
Cerulenin:
0μM
4.0μM
WT
sec14-1ts
esa1-L254Pts
sec14-1ts esa1-L254Pts
30°C
32°C
33.5°C
37°C

Figure S6. Inhibition of fatty acid biosynthesis causes a synthetic growth defect in *sec14-1^{ts}* suppressed by NuA4 mutants *eaf7Δ* and *esa1-L254P^{ts}*. Inhibition of fatty acid biosynthesis by cerulenin treatment causes synthetic growth defect with *sec14-1^{ts}* suppressed by *EAF7* and *ESA1* mutants. Wildtype (YKB1079), *sec14-1^{ts}* (YKB3144), *sec14-1^{ts}eaf7*Δ (YKB4068), *esa1-L254P^{ts}* (YKB4236), and *sec14-1^{ts}esa1-L254P^{ts}* (YKB4242) cultures were grown in YPD at 30°C prior to being diluted to an OD_{600} of 0.1 and 4 times 10-fold serial dilutions were plated on YPD with indicated amounts of cerulenin for two days at 30°C, 32°C, 33.5°C, and 37°C. Images are representative of three biological replicates.
